# Modeling *Lactococcus lactis *using a genome-scale flux model

**DOI:** 10.1186/1471-2180-5-39

**Published:** 2005-06-27

**Authors:** Ana Paula Oliveira, Jens Nielsen, Jochen Förster

**Affiliations:** 1 Fluxome Sciences A/S, Søltofts Plads, Building 223, DK-2800 Kgs. Lyngby, Denmark; 2 Center for Microbial Biotechnology, BioCentrum-DTU, Technical University of Denmark, Building 223, DK-2800 Kgs. Lyngby, Denmark

## Abstract

**Background:**

Genome-scale flux models are useful tools to represent and analyze microbial metabolism. In this work we reconstructed the metabolic network of the lactic acid bacteria *Lactococcus lactis *and developed a genome-scale flux model able to simulate and analyze network capabilities and whole-cell function under aerobic and anaerobic continuous cultures. Flux balance analysis (FBA) and minimization of metabolic adjustment (MOMA) were used as modeling frameworks.

**Results:**

The metabolic network was reconstructed using the annotated genome sequence from *L. lactis ssp. lactis IL1403 *together with physiological and biochemical information. The established network comprised a total of 621 reactions and 509 metabolites, representing the overall metabolism of *L. lactis*. Experimental data reported in the literature was used to fit the model to phenotypic observations. Regulatory constraints had to be included to simulate certain metabolic features, such as the shift from homo to heterolactic fermentation. A minimal medium for *in silico *growth was identified, indicating the requirement of four amino acids in addition to a sugar. Remarkably, *de novo *biosynthesis of four other amino acids was observed even when all amino acids were supplied, which is in good agreement with experimental observations. Additionally, enhanced metabolic engineering strategies for improved diacetyl producing strains were designed.

**Conclusion:**

The *L. lactis *metabolic network can now be used for a better understanding of lactococcal metabolic capabilities and potential, for the design of enhanced metabolic engineering strategies and for integration with other types of 'omic' data, to assist in finding new information on cellular organization and function.

## Background

Lactic acid bacteria (LAB) are a heterogeneous group of microorganisms able to convert carbohydrates into lactic acid. They are applied worldwide in the industrial manufacture of fermented food products, mainly in the dairy industry. During fermentation LAB primarily produce lactic acid from the available carbon source, resulting in the rapid acidification of the food raw material, which is an important parameter in the preservation and extension of shelf life of food products. LAB metabolism also contributes for the development of desired product properties such as flavor and texture [1,2]. Because of their long tradition of safe use (GRAS microorganism), their capacity to grow rapidly on lactose-based media derived from milk and their potential to generate a variety of metabolic products, LAB also have the potential to be used as cell-factories in bioreactors for the *in situ *production of compounds that contribute to the flavor, texture or health benefits of foods [1].

Among LAB, *Lactococcus lactis *is, by far, the most extensively studied with respect to its physiology, metabolic pathways and regulatory mechanisms, and its genome was the first LAB genome to be completely sequenced and annotated [3]. Lactococci are nutritionally fastidious organisms with a very limited biosynthetic capacity. Anabolic precursors are primarily imported from the growth media, whereas only a minor fraction is synthesized *de novo *from a carbon source. The major part of the carbon from carbohydrates is converted into fermentation end-products. For example, during growth on glucose, only about 5% of the metabolized sugar is converted into biomass [4]. The very limited biosynthetic capacity of *L. lactis *implies that, for optimal growth, they require exogenous supply of a fermentable sugar, numerous vitamins and amino acids, phosphate, potassium and magnesium [5].

*L. lactis *is a facultative anaerobic bacterium. Some strains are capable of growing in the presence of oxygen and adjust their metabolism accordingly, while others are strongly inhibited under aerobic conditions. As this microorganism lacks a functional respiratory chain, the ability to grow aerobically has been linked to the presence of NADH-oxidases. Since *L. lactis *is not able to perform respiration, ATP is only formed through substrate level phosphorylation. Thus, in order for Lactococci to grow at a high specific growth rate, a high carbohydrate degradation rate (and, concomitantly, an efficient sugar transport system) is called for. The main function of the sugar metabolism in *L. lactis *is to generate the energy necessary for rapid growth and for maintenance of intracellular pH during acidification of the environment [6].

Due to its major importance as a laboratorial and industrial microorganism, and because of its relatively simple metabolism and limited biosynthetic capabilities, *L. lactis *has been an organism of choice for many metabolic engineering purposes [1,6,7]. Hence, the design of enhanced metabolic engineering strategies calls for models where cellular behavior can be predicted.

The reconstruction of the entire metabolic reaction network of a cell and subsequent application of genome-scale flux models has been conducted for many organisms, including bacteria, yeast, fungi and animal cells [8-12]. These models have the potential to become common modeling tools. One approach that has been used to explore the capabilities of these large metabolic networks is flux balance analysis (FBA). This is simply a linear programming posed problem in which the constraints are defined by the stoichiometry of enzymatic reactions and transport steps. A solution to the problem, i.e., a set of fluxes through all the defined reactions, can be found through specifying an objective function, which is often defined as the optimization of a certain flux of interest, e.g., the flux towards formation of biomass. Maximization of biomass production has been shown to allow description of overall metabolic behavior in a number of cases, probably because most cells have evolved, under laboratory conditions, towards the maximization of their growth performance [13]. By using appropriate constraints and a meaningful objective function, FBA has been successfully used in exploring the relationship between genotype and phenotype and in the prediction of product yields and growth rates under changing environmental and genetic conditions, at steady state [14-16]. More recently, another approach has been proposed for dealing with the effect of gene deletions in the prediction of flux distributions, based on quadratic programming [17]. This minimization of metabolic adjustment (MOMA) approach relies on the assumption that optimal growth may initially not be true for mutants generated artificially in the laboratory as usually those mutants have not yet undergone evolution towards optimal growth.

In this work the reconstruction process of the metabolic network of *L. lactis *is presented. Network reactions were collected using the annotated genome sequence, biochemical and metabolic pathways databases, biochemistry books and journal publications. Once the network was established, FBA and MOMA were applied to analyze the network capabilities and to model phenotypic behavior under anaerobic and aerobic conditions. Simulation results were compared with experimental data available in the literature. Furthermore, the model was used to identify possible metabolic engineering targets to design an efficient diacetyl producing strain.

## Results and discussion

### Characteristics of the reconstructed network

The reconstruction process resulted in a network comprising 621 reactions and 509 metabolites. The entire reaction database can be consulted in the Additional file 1 and is also available at . A total of 358 ORFs out of the detected 2310 ORFs in the sequenced genome were considered, corresponding to 476 associated reactions. The remaining 145 reactions were included based on biochemical/physiological considerations or inferred by the demands imposed on the metabolic network. Namely, reactions were included based on experimental information reported in literature to account for pathway gaps, transport steps and biomass assembly. From the entire set of reactions, 492 correspond to intracellular reactions while 129 are transport fluxes over the cytoplasmic membrane. From the 358 ORFs considered, 63 ORFs account for genes that have been isolated and identified from *L. lactis*, 286 ORFs have high homology with identified genes from other organisms, 2 ORFs are annotated sequences derived from low level homologues and 7 ORFs are described as probable homologues of unknown function (ORF reliability classification based on [18]). The reaction database includes a total of 509 network metabolites, from which 414 are intracellular and 95 are external metabolites. 87 out of these 95 are metabolites secreted or taken up by the cell without undergoing phosphorylation, and so it can be pointed out that the reconstructed network accounts for 422 unique metabolites. Table 1 summarizes the main characteristics of the reconstructed metabolic network of *L. lactis*.

#### Network reactions

From the 358 ORFs considered in the reconstruction process, ORFs that are assigned to energy metabolism, amino acid biosynthesis and nucleotides metabolism account for more than half of the total number. These ORFs are involved in 476 reactions. The relatively high number of associated reactions is mainly due to the existence of low substrate-specific enzyme activities catalyzing different reactions. For instances, the model includes a low-specific aminotransferase (araT) involved in the metabolism of several amino acids and defined to catalyze 18 reactions in the model. An equation on biomass formation was developed to account for the drain of precursors and building blocks into biomass. A detailed description of its assembly can be found in the Additional file 2. This equation also accounts for energy requirements associated with growth, which have been estimated through model fitting against experimental observations as being 18.15 mmol ATP gDW^-1^. One reaction was included to account for non-growth dependent ATP maintenance. For growth under glucose limitation this value has been previously experimentally determined and equals 1 mmol ATP gDW^-1^h^-1 ^[19].

#### Network metabolites

The metabolic network of *L. lactis *contains 509 metabolites, 422 of which are unique metabolites. It is through metabolites that reactions are connected, as the product of one reaction becomes the substrate of another. At a zoom-out level, different biochemical pathways within a cell are interconnected by virtue of metabolites that participate in more than one pathway [20]. In particular, cofactors like ATP, NADH and NADPH play an important role in connecting the many different pathways. The most frequent metabolic intermediates in the reconstructed network are presented in Table 2. A total of 28 intracellular metabolites were not connected into the overall metabolic network. These non-connected metabolites take part in 25 "non-connected" reactions, catalyzed by 21 "non-connected" gene products (see Additional file 3)

### Growth requirements and minimal media

*L. lactis *is able to metabolize a variety of different sugars and other carbon sources [2,21] to obtain energy, redox power and precursor metabolites for macromolecular biosynthesis. Fermentable carbon sources utilized by *L. lactis *include fructose, galactose, glucosamine, glucose, lactose, maltose, mannitol, mannose, ribose, sucrose and trehalose.

The capabilities of the *in silico *model to utilize different carbon sources to grow were evaluated using FBA, using comparable uptake rates and carbon-limited conditions. All the above mentioned carbon sources were supplied, one at a time, together with all amino acids and nucleotides. A sugar consumption rate of 13.6 mmol gDW^-1 ^h^-1 ^was considered (6.8 mmol gDW^-1 ^h^-1 ^in the case of disaccharides), both under aerobic and anaerobic conditions.

The specific growth rate under anaerobic conditions was always higher than at aerobic conditions when the specific oxygen consumption was set to 3.61 mmol gDW^-1 ^h^-1 ^[22], which is in good agreement with experimental evidence [23]. The predicted anaerobic specific growth rate on glucose was 0.79 h^-1^, while the aerobic specific growth rate was 0.62 h^-1^. In case oxygen uptake is not constrained (unlimited oxygen uptake is allowed) the specific growth rate was 0.82 h^-1^. Constrained aerobic growth is slightly lower than anaerobic growth due to the different flux constraints applied to the enzymes that metabolize pyruvate into acetyl-CoA (see Methods). Pyruvate-formate lyase is only active under anaerobic conditions, while the pyruvate dehydrogenase (PDH) complex is only active during aerobic growth [24]. This forces the cell to produce more NADH under aerobic conditions, since the PDH complex is a NADH producing step and is also an essential reaction for formation of the precursor metabolite acetyl-CoA. However, model results indicate that for low oxygen consumptions there is a limited capacity to recycle NADH through NADH-oxidase, therefore causing the cell to reduce the flux through the PDH complex, decreasing the amount of acetyl-CoA available for lipid metabolism and, consequently, for biomass formation.

Growth on mannose, galactose, sucrose, lactose and glucosamine was found to be the same as for growth on glucose. The capacity to grow on trehalose and maltose was slightly higher, due to a decreased ATP requirement for the synthesis of the corresponding phosphorylated sugars. Anaerobically, growth on fructose and mannitol led to a decrease of 3% and 16% in biomass formation, respectively, compared with growth on glucose. This decrease has been qualitatively observed experimentally [21,25,26]. In the case of fructose, the difference seems to be associated with a lower capacity to generate NADPH due to a lower flux through the pentose phosphate pathway. In the case of mannitol, the lower biomass formation rate seems to be related both with a decrease in the capacity of generating NADPH and with a NADH burden due to formation of an additional NADH molecule during the reaction catalyzed by mannitol-1-phosphate dehydrogenase. Probably as consequence of that burden, the simulated growth leads to the production of high amounts of ethanol and formate, which is in good agreement with experimental results reported by Neves *et al*. (2002) [26].

In addition to a carbohydrate source, the minimal amino acid requirements were determined by omission of each amino acid at a time. The single omission of arginine, methionine and valine was found to prevent *in silico *growth, even when all other amino acids are supplied and despite the presence of the biosynthetic pathways for these amino acids in the reconstructed network. In the case of valine this result is trivial, since simulations were run under the constraint that the reaction catalyzed by bcaT_1 can only occur in the catabolic direction, and there is no alternative pathway for valine synthesis. In the case of methionine, the reason appears to be the lack of available FAD (cofactor) to sustain growth. Finally, if no arginine is supplied, the linear problem becomes infeasible. Nevertheless, arginine synthesis is observed when maximizing for an arginine drain.

Growth was not observed when only these three amino acids were supplied. If glutamate (or, alternatively, glutamine) is allowed to be taken up in the presence of these three amino acids, growth occurs. Therefore, glucose, arginine, methionine, glutamate (or glutamine) and valine were found to be the minimal required medium for growth. *L. lactis *strains are usually auxotrophic for 7 to 9 amino acids, including these four [5,27]. Growth in the above defined minimal media is, however, 54% lower than in rich media. When all the amino acids reported as essential for *L. lactis *IL1403 [27] are allowed to be taken up by the model, growth rate increases to 87% of the value in rich media. This corresponds to the addition of asparagine, histidine, isoleucine and leucine to the above defined minimal media.

### Single gene deletion analysis

Single gene deletion analyses were computed to predict the lethal effect of deleting each individual gene. Prediction capability was assessed by comparing simulation results with experimental data on gene lethality reported for *L. lactis *[28] and *Bacillus subtilis *[29], a related Gram-positive bacteria of low G+C content, during growth in rich media. 25 genes were found in literature as being lethal, out of the 34 predicted by the model. From these, one is a false lethal (ThyA), while no references were found for the remaining 8 (see Additional file 4). Regarding these analyses, it has to be point out that regulatory proteins and constrains were seldom included, so it is likely that we are underestimating the number of actual lethal genes.

The effect of the deletion of each reaction was also assessed. These computational studies were performed both in rich media and minimal media, and both under aerobic and anaerobic conditions. FBA and MOMA were used to compute these simulations, with MOMA predicting around 10 more lethal genes/reactions than FBA (Table 3). We focus the following analysis on MOMA results (all details can be found in the Additional file 4). The deletion of 24.6% of the genes showed to be lethal in minimal media, while in rich media this number decreases to 12.1% (percentage based on the number of ORFs included in the model, discounting the 21 "non-connected" gene products). The single deletion of reactions accounts for 23.1% lethal reactions in minimal media and 14.1% in rich media (considering 596 network reactions: all the 621 network reactions minus the 25 "non-connected" reactions). Additionally, it was observed that although the number of lethal reactions is always higher than the number of lethal genes, this is mainly due to the existence of reactions catalyzed by unknown gene products (enzymes without a corresponding annotated ORF).

Aerobic and anaerobic simulations led to similar results, with a few more genes/reactions being lethal under aerobic conditions, namely those associated to oxygen utilization, CO_2 _production and to the pyruvate dehydrogenase (PDH) complex. Under aerobic conditions, the PDH complex is the only pathway leading to the formation of acetyl-CoA from pyruvate, and as acetyl-CoA is an essential metabolite in many processes (such as lipid formation), deletion of the PDH complex results in a lethal phenotype.

When comparing the growth capabilities in both rich and minimal media, it can be observed that approximately 40 additional genes are lethal during growth in minimal media. These are mainly genes associated with biosynthesis of amino acids. From the 83 lethal genes in minimal media, 19 encode for gene products that catalyze more than one reaction. Only one of these 83 genes encoded for a protein that has an isoenzyme.

### Modeling the shift from homolactic to heterolactic metabolism

To further evaluate the modeling capabilities of FBA we used the genome-scale flux model to simulate the shift from homolactic to heterolactic metabolism in *L. lactis *growing anaerobically. This process consisted in comparing simulation results with experimental observations reported by Thomas, T.D. *et al*. (1979) [30] for glucose limited anaerobic chemostat cultures, though refining and tuning the model through the introduction of appropriate biological meaningful constrains. Nevertheless, one should notice that although FBA is based on steady-state assumption and therefore is more suitable for simulation of metabolic behavior in continuous cultivations, model results can also be compared with batch experimental data under the assumption of pseudo steady-state during the exponential growth phase.

Experimental observations reported in literature suggest that, for *L. lactis*, product formation at high dilution rates during continuous cultivations is similar to product formation during batch growth at high glucose concentrations, resulting in lactic acid as the sole metabolic product [19,31]. On the other hand, growth at low dilution rates in continuous conditions or at low concentrations of glucose in batch conditions (ie, in the presence of low fluxes through glycolysis) results in a mixed-acid fermentation, where formate, ethanol and acetate are produced in a molar ratio of 2:1:1 [32]. While indicating that the shift in metabolism is due to regulation, these observations also suggest that different flux constraints would have to be introduced to model the shift from homolactic to heterolactic metabolism.

For simulations of phenotypic behavior, the first obvious objective function was thought to be "maximization of growth" while constraining for substrate uptake. However, it became clear that constraining values for amino acid uptake rates would pose a problem, as the *in silico *strain was auxotrophic for some amino acids, which could act as a carbon source, and no information was found available in the literature for amino acid uptake rates under chemostat conditions. To solve this problem, consumption rates were approximated to be equal to the amino acids requirement for biomass formation. In the absence of experimental information for amino acid uptake rates another approach was followed, in which phenotypic behavior was simulated by constraining the growth rate and minimizing for substrate uptake. Both approaches led to different and complementary qualitative results regarding the network capabilities, as discussed below.

#### Maximizing for growth

When maximizing for biomass formation, the metabolic phenotype cannot be correctly predicted from the reconstructed network by simply stating anaerobic constraints (see Methods). The process of tuning and refining the model is discussed below and detailed results are summarized in Figures 5 and 6 (see Additional file 5). Simulations in which the specific consumption rate of glucose was constrained to values equal or greater than 14.1 mmol gDW^-1 ^h^-1 ^revealed the occurrence of multiple solutions for products of metabolism. From these simulations it was observed that growth rate does not change with different glucose uptake rates, indicating that growth is nitrogen-limited and reaches a maximum at 0.82 h^-1 ^(in good agreement with the fact that amino acid uptake rates were established based on the amino acid cell content at 0.8 h^-1^), while for glucose uptake rates lower than 14.1 mmol gDW^-1 ^h^-1 ^growth is glucose-limited. Since lactate production is reported under high glycolytic fluxes, and this is known to be due to regulation exerted by the NADH/NAD^+ ^ratio on the pyruvate-formate lyase [33], additional constraints can be included in the model in order to account for regulatory information. For example, Covert, M.W. *et al *[34] described a Boolean on/off approach to account for regulation within the metabolic network. However, since pyruvate-formate lyase is essential to the formation of acetyl-CoA (and therefore, biomass) under anaerobic conditions, a simple on/off Boolean statement cannot be applied. Therefore, based on fitting the model against experimental data, the pyruvate-formate lyase flux was set to 2.15 and 9.8 mmol gDW^-1 ^h^-1 ^to simulate homolactic growth when glucose uptake rates equal 24.6 and 18 mmol gDW^-1 ^h^-1^, respectively.

In all simulations it can be observed that the metabolic model predicts trace production of amino acid catabolism products (3-methyl-2-oxobutanoate, 3-methyl-2-oxopentanoate, 4-methyl-2-oxopentanoate, phenyllactate, methional), which is in good agreement with experimental results [35-37]. Interestingly, it can be observed that although the formation of these catabolic products is not directly accompanied by the formation of energy or reduced compounds, they all contribute to a gain in biomass formation.

**Figure 1:**
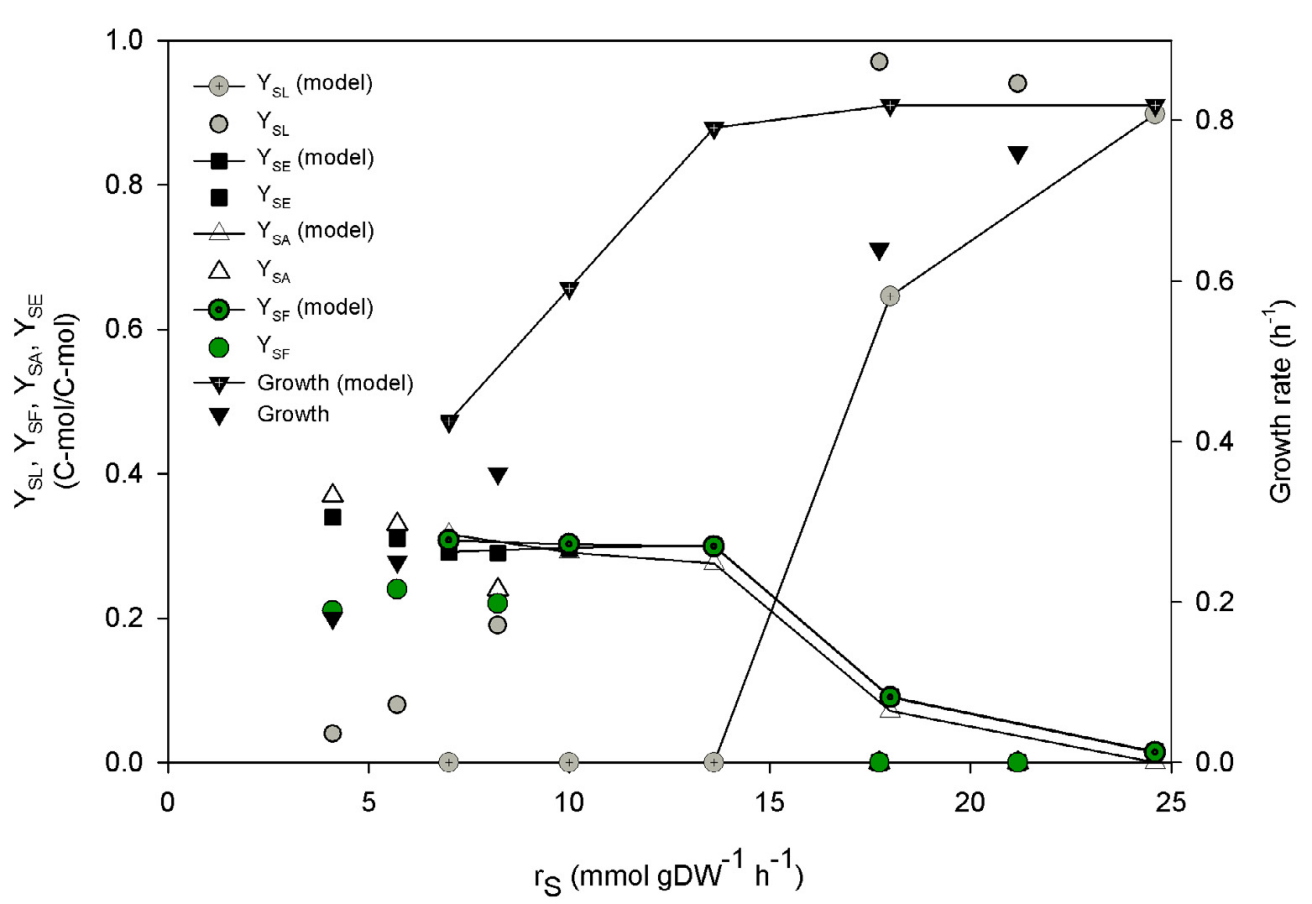
**Plotting model and experimental data for anaerobic growth, chemostat conditions, when maximizing for biomass formation**. Growth rate and conversion yields for lactate (L), formate (F), ethanol (E) and acetate (A) on glucose (S) are plotted against glucose uptake rate. Model results are from simulations NX5 to NX10 (see Table 5 in Additional file 5). Experimental data is from Thomas, T.D., *et al *(1979) [30]. Ethanol and formate predictions are overlapped.

Plotting experimental against model results for biomass, lactate, formate, ethanol and biomass formation (Figure 1) shows that simulations can reproduce the general observed tendencies for product and biomass formation, overestimating biomass formation. Also formate, ethanol and acetate are slightly overestimated for the range of the simulated conditions. At high glucose uptake rates, the model cannot predict the absence of these products. From Figure 1 it can further be observed that lactate production is poorly described with the considered constraints. Additional constraints could have been introduced to better describe lactate formation. However, this imposes too many uncertain variables, namely for amino acid consumption.

#### Minimizing for substrate uptake

To overcome this difficulty another approach was used: the minimization of substrates uptake while constraining the growth rate. From simulation results, the formation of end-products of the pyruvate metabolism is observed. No by-products from the amino acid catabolism were predicted using this approach, as it minimizes amino acids uptake, and therefore no excess of amino acids is available for catabolism of amino acids. Without further constraints, mixed-acid fermentation is observed, as this is energetically more favorable for the cell (more ATP is formed when acetate is synthesized). However, as mentioned above, high fluxes through glycolysis lead to regulation effects towards homolactic fermentation. Consequently, the reaction catalyzed by pyruvate-formate lyase was constrained to values between 0 and 9 mmol gDW^-1 ^h^-1 ^(see Table 6) by fitting simulation results against experimental data

**Figure 2:**
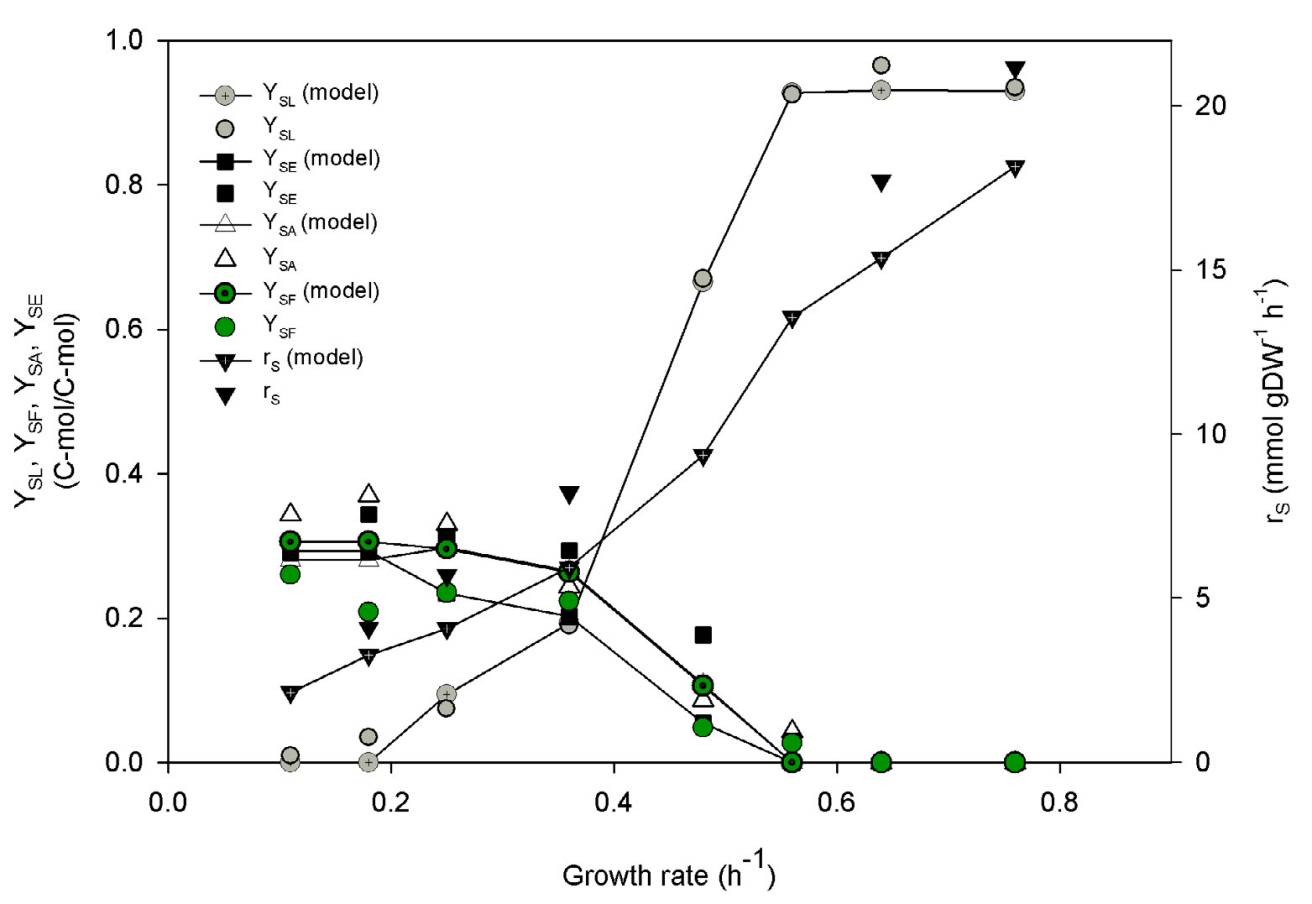
**Plotting model and experimental data for anaerobic growth, chemostat conditions, when minimizing for substrates uptake**. Glucose uptake rate and conversion yields for lactate (L), formate (F), ethanol (E) and acetate (A) on glucose (S) are plotted against growth rate. Model results are from simulations NS3, NS5, NS7, NS8 to NS11 (see Table 6 in Additional file 5). Experimental data is from Thomas, T.D., *et al *(1979) [30]. Acetate and formate predictions are overlapped.

From Figure 2 it is observed that model results for formate, ethanol and acetate formation fairly fit experimental observations, although the model underestimates ethanol and acetate production and overestimates formate production. Furthermore, glucose consumption is also underestimated by model predictions. The difference is around 14% at high substrate uptake rates and 25% for lower values. This can be due, for example, to differences in the maintenance energy between the real cell and the simulated system.

**Figure 3:**
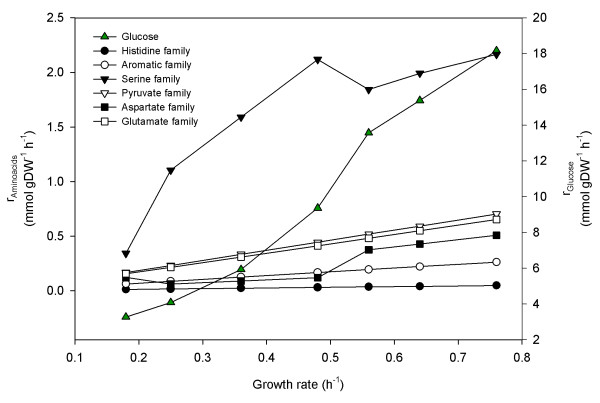
**Model predictions for glucose and amino acid uptake rates versus the growth rate. **Model results are from simulations NS3, NS5, NS7, NS8 to NS11. Amino acids were grouped into six families: *Histidine *(His), *Aromatic *(Phe, Trp, Tyr), *Serine-family *(Cys, Gly, Met, Ser, Thr), *Pyruvate-family *(Ala, Ile, Leu, Val), *Aspartate-family *(Asp, Asn, Lys) and * Glutamate-family *(Arg, Glu, Gln, Pro) [47].

Analysis of amino acid requirements for *in silico *growth interestingly shows that, for most of the cases, amino acid uptake rates linearly increase with the growth rate (Figure 3). This observation only applies if metabolic families of amino acids, derived from the same precursor, are considered. Threonine was included in the serine-family (instead of the expected aspartate-family), as it is related with glycine through the reaction L-threonine <-> glycine + acetaldehyde. Even with this change, linearity is not observed for both these families. Two reasons can be hypothesized to explain that: either threonine synthesis pathway depends on the growth rate or, alternatively, it is the serine contribution for the pyruvate pool that depends on biomass formation rate.

When minimizing for substrate uptake, the predicted amino acid uptake rates corresponds to the theoretical amino acid requirements for the cell to grow at the established growth rate. However, *in vivo *amino acids consumption is usually higher than the theoretical needs for macromolecules biosynthesis. The excess of amino acids can then be further catabolized, resulting in the secretion of amino acid by-products.

### Amino acid biosynthesis capabilities

In all simulations under the objective of minimizing the substrate uptake, it could be noted that some amino acids are not taken up from the medium even if they are present in the medium. The cell seems to prefer to synthesize some of them. Amino acids completely synthesized by the *in silico *strain were alanine, aspartate, glycine and phenylalanine (either if glutamate is or is not supplied). Remarkably, this observation is in very good agreement with experimental data reported by Jensen, N.B. *et al*. (2002) [38] for *L. lactis *subsp *cremoris*. In their work, they analyzed the capacity for the *de novo *biosynthesis of amino acids when all amino acids except glutamate were supplied, having observed that alanine, aspartate, phenylalanine and threonine, were synthesized *de novo *by the cell. Analyzing the flux distribution of the reactions involved in amino acid biosynthesis, it can be found that those preferences are associated with an increased production of 2-oxoglutarate, leading to an increased formation of L-glutamate.

### Identfication of metabolic engineering targets

A relevant application of genome-scale metabolic models is the simulation of cellular behavior in response to genetic perturbations. Namely, genome-scale metabolic models can be used as tools in the design of metabolic engineering strategies, aiming at finding genetic targets leading to enhanced desired properties [20]. We exemplify here the use of the reconstructed network to predict potential ways to increase the yield of diacetyl, an important flavor compound in dairy products. Diacetyl is a by-product of *L. lactis *fermentative metabolism and it is produced chemically by oxidative decarboxylation of the metabolic intermediate 2-acetolactate (which is derived from the condensation of two molecules of pyruvate). Hence, knockout strategies leading to an increased yield of 2-acetolactate in glucose were investigated using FBA and MOMA.

**Figure 4:**
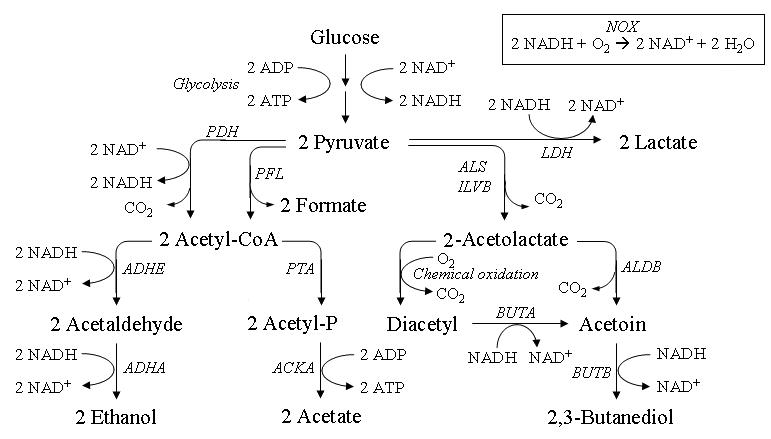
**The pyruvate metabolism of *Lactococcus lactis***. LDH: lactate dehydrogenase; PDH: pyruvate dehydrogenase complex; PFL: pyruvate formate-lyase; ADHE: acetaldehyde dehydrogenase ; ADHA: alcohol dehydrogenase; PTA: phosphotransacetylase; ACKA: acetate kinase, ALS/ILV B: catabolic and anabolic 2-acetolactate synthase; ALDB-acetolactate decarboxylase; BUTA-diacetyl reductase; BUTB: acetoin reductase; NOX: NADH – oxidase. ..

Lactococci metabolism around pyruvate is depicted in Figure 4. From pyruvate, carbon can be redirected towards acetyl-CoA, lactate or 2-acetolactate. Common strategies to increase the flux towards 2-acetolactate have been gene knockouts around pyruvate (except of the lethal pyruvate dehydrogenase complex), over-producing the 2-acetolactate synthase and/or over-producing an heterologous NADH-oxidase [1,7,39-41]. For example, Henriksen *et al *(2001) [24] have succeed to convert up to 95% of glucose towards the formation of 2-acetolactate and related compounds through the deletion of lactate dehydrogenase and pyruvate-formate lyase. Hugenholtz *et al *(2000) [39] constructed a high-producing diacetyl strain able to redirect 80% (16%) of glucose into 2-acetolactate (diacetyl), by deleting acetolactate synthase and over-expressing an heterologous NADH-oxidase.

The first step in our modeling strategy was to find a set of appropriate constraints that lead to results comparable with experimental observation. By simply allowing unconstrained uptake of oxygen it was possible to obtain both 2-acetolactate and acetate as the main by-products of metabolism, which is in good agreement with experimental reports [39]. Oxygen uptake was about 1.4 mmol O_2 _/ mmol glucose. The simulated growth rate and yield of 2-acetolactate on glucose are presented in Table 4. However, a plurality of solutions were observed under the chosen conditions. In order to minimize the number of solutions, we constrained the activity of lactate dehydrogenase (ldh), 2-acetolactate synthase (aldB) and alcohol dehydrogenase (adhA) to zero. This forces pyruvate to be redirected either towards acetate or 2-acetolactate (and related compounds, ie, C4 and C5 products).

Next, the impact of single gene deletions on product formation was simulated by maximizing for growth. It was found that the deletion of *PTA *leads to a slight yield increase. The enzyme Pta catalyzes the conversion of acetyl-CoA to acetyl-P, and its deletion eliminates the production of acetate. Furthermore, another single gene deletion was run on the "mutant" Δ*ldh*Δ*ald*Δ*adhA*Δ*pta *and three deletions leading to a higher production of 2-acetolactate were found, Δ*FHS*, Δ*SERA *and Δ*ZWF*. The enzyme Fhs is a formyltetrahydrofolate synthetase (EC 6.3.4.3), involved in the folate metabolism. Its contribution towards a higher yield of 2-acetolactate is due to an increase in carbon availability for by-product formation, since this reaction is used by the cell to generate ATP and formate (in rich media). The enzyme SerA, a 3-phosphoglycerate dehydrogenase, is involved in serine metabolism. This part of the metabolism connects with Fhs. Hence, this deletion increases 2-acetolactate yield similarly to the deletion of *SERA*. Finally, *ZWF *deletion is only predicted by MOMA as being advantageous. *ZWF *encodes for glucose-6-phosphate 1-dehydrogenase, the first step of the pentose-phosphate pathway. This deletion is accompanied by a decrease in biomass formation, because the availability of NADPH decreases. Therefore, more carbon is redirected towards other products of metabolism (C4 and C5 compounds), resulting in an increase of 2-acetolactate yield. Observed yields and biomass formation rates are summarized in Table 4. One should keep in mind that these are theoretical maximum specific growth rates at which high yields of 2-acetolactate can be obtained. Other factors such as a limitation in the uptake rate of oxygen may lead to experimentally lower values.

## Conclusion

The metabolic network of *Lactococcus lactis *was reconstructed based on genomic, physiological and biochemical information, comprising a total of 621 reactions and 509 metabolites. Lactococcal network characteristics are comparable with other bacterial genome-scale reconstructed networks.

Metabolic network analysis was carried out using FBA and MOMA. The genome-scale metabolic model for *L. lactis *was shown to be robust and able to predict many experimental observations, when considering additional constraints derived from available experimental data. The model proved to be a useful tool to analyze the metabolic capabilities of *L. lactis *and to understand how the individual components in the system interact and influence the overall cell function. For example, the model could predict that, if all the amino acids were supplied, the cell will prefer to synthesize *de novo *alanine, aspartate, glycine and phenylalanine. The model can now be used as a useful tool to test or develop novel metabolic engineering strategies to redirect fluxes towards the production of important products such as diacetyl, alanine and exopolysaccharides [1].

Reconstructed metabolic networks are finding many other applications than the ones described in this work. Optimization methods have also been used to assess maximum capabilities of the network and to analyze gene dispensability [42]. More recently, different methods to integrate metabolic networks with transcriptome data were described [43-45]. Patil and Nielsen (2005) [45] describe a method that represents the metabolic network as an enzyme-metabolite interaction graph and, assigning expression scores to each enzyme, it is possible to highlight which metabolites are more affected by transcriptional changes (the so-called reporter metabolites). The method also identifies the most active metabolic sub-network responding to a particular perturbation. In the near future it is expected that metabolic networks will be further used together with other types of 'omic' information and help to reveal hidden information on cellular organization and function.

## Methods

### Network reconstruction

The reconstruction process of the metabolic network of *L. lactis *involved a comprehensive search of the current knowledge of its metabolism. The process started based on the ORFs information from the annotated genome of *L. lactis *IL 1403 [3]. From this list, a reaction database was built using the available genomic, biochemical and physiological data accessible in databases and relevant literature. Particular focus was given to the reactions reversibility. Reactions whose reversibility could not be assessed were defined as reversible. The reaction set also includes a reaction for biomass formation defined as a drain of major building blocks into biomass. One reaction was included to account for non-growth dependent ATP maintenance. After the initial assembly of the entire metabolic network, the list was re-examined to account for metabolic and physiological details. The list of reactions was manually and carefully examined regarding reliability of gene assignment, all possible/probable catalytic activities of gene products and the *in vivo *reaction reversibility.

### Biomass composition

An equation for biomass formation was developed to account for the drain of precursors and building blocks into biomass. Biomass synthesis was set as a linear combination of seven macromolecular components – proteins, DNA, RNA, lipids, lipoteichoid acids, peptidoglycan and polysaccharides – which were considered to account for the cell overall biomass composition. The individual composition of every component was maintained at a fixed stoichiometry, independent of the specific growth rate. Cellular energy requirements were also considered, by taking into account information on the ATP cost of polymerisation and growth and non-growth associated ATP maintenance. The detailed calculation of the biomass composition can de found in the Additional file 2.

From the developed equations, an elemental biomass composition was determined for the reconstructed *in silico *strain, CH_1.95_O_0.63_N_0.22_P_0.02_S_0.01, _corresponding to a molecular weight of 27.8 g/C-mol. This is in good agreement with values found in the literature. Novak, L. *et al*. (2000) have measured a value of 27.7 g/C-mol for *L. lactis*.

### Mathematical frameworks: FBA and MOMA

Flux balance analysis (FBA) is a linear programming posed problem where equations are defined by mass balance-derived stoichiometric reactions, constrains are imposed by fluxes limitations and the objective function is established based on a biological meaningful objective.

Given a stoichiometric matrix derived from mass balance around all metabolites of a cell and assuming steady state, a system of linear equations is produced which simply states that the incoming fluxes are balanced by the outgoing fluxes. For a metabolic network comprising *N *metabolites and *M *metabolic reactions, the stoichiometric matrix can be written as:



where *S*_*ji *_is the stoichiometric coefficient of metabolite *j *in reaction *i*, *v*_*i *_represents the flux of reaction *i*, and *b*_*j *_quantifies the network's uptake (or secretion) of metabolite *j*. Reversible reactions are defined simply as two irreversible reactions in opposite directions, constraining all fluxes to nonnegative values, *ie*:

*v*_*i *_≥ 0, ∀ *i *    (2)

Some other constraints based on physiological or physicochemical aspects may be applied, such as thermodynamics considerations, regulation effects and maximal enzymatic rates:

*α *≥ *v*_*i *_≥ *β*_*i*_, ∀ *i *    (3)

Establishing a particular objective function, *Z*, written as a linear combination of existing variables, the optimal solution can then be found at one corner of the set of feasible solutions. For metabolic applications, typical objective functions are maximization of biomass formation or minimization of substrate consumption.

Flux balance analysis can then be simply summarized as a linear programming problem posed as:

maximize *Z = eg, biomass formation *    (4)

subject to



*v*_*i *_≥ 0, ∀ *i*

*α *≥ *v*_*i *_≥ *β*_*i*_, ∀ *i*

Another mathematical framework that can be used to find flux distribution solutions is the so-called minimization of metabolic adjustment (MOMA) [17]. MOMA relaxes the assumption of optimal growth flux for gene deletions, displaying a suboptimal flux distribution that is intermediate between the wild-type optimum and the mutant optimum. The philosophy of MOMA can be interpreted as the projection of the FBA optimum onto the feasible space of the mutant. Therefore, MOMA can be posed as a quadratic programming problem, with the same set of linear constrains as for FBA and where the objective function is to minimize the distance between the feasible solutions space of both wild-type and mutant.

Both the FBA and the MOMA problems were solved with an in-house developed software using the following solvers: GNU Linear Programming Kit  and Object-Oriented software for Quadratic Programming .

### Model constraints

All simulations were run allowing free uptake of nucleotides (xanthine, uracil, cytosine, adenine, guanine and hypoxanthine), phosphate, biphosphate and sulfate. Additionally, non-growth associated ATP requirement was always set to 1 mmol ATP gDW^-1 ^h^-1^, which is the value experimentally estimated for carbon-limitated chemostat cultures [19].

To simulate growth under anaerobic conditions, the oxygen uptake rate was set to zero. Experimental insight on pyruvate metabolism led to two additional constraints. Since it is known that the pyruvate dehydrogenase (PDH) complex is not active in the absence of oxygen [46], pdhA_1 and pdhB_1 fluxes were therefore set to zero.

Aerobic growth was simulated by allowing oxygen to be taken up by the model. Unless otherwise stated, the oxygen consumption was set to 3.61 mmol gDW^-1 ^h^-1 ^[22]. Under aerobic conditions the pyruvate dehydrogenase is active but the pyruvate-formate lyase (PFL) is strongly inhibited by oxygen [24]. pdhA_1 and pdhB_1 were therefore left unconstrained while the pfl_1 flux was set to zero.

### Evaluation of growth requirements and minimal media

Network capabilities to utilize different sugar sources were evaluated using FBA. A number of different sugars were supplied, one at a time, together with all amino acids and nucleotides. A sugar consumption rate of 13.6 mmol gDW^-1 ^h^-1 ^was allowed, both under aerobic and anaerobic conditions (6.8 mmol gDW^-1 ^h^-1 ^in the case of the disaccharides sucrose, lactose, maltose and trehalose). When the specific growth rate was higher than 0.01 h^-1^, the sugar was considered to be used for growth. This value was established based on the observation that when supplying only amino acids but no sugar, a specific growth rate of 0.01 h^-1 ^is predicted by the model, which is not observed experimentally [19].

Amino acid requirements for *in silico *cell growth were analyzed under anaerobic conditions. The dilution rate was fixed to 0.18 and 0.76 h^-1 ^and the objective function used in this investigation was the minimization of substrates uptake rate. Beginning with all the amino acids available, single-omissions were simulated by setting the corresponding uptake rate to zero, one at a time. If no biomass is formed, the omitted amino acid was defined as essential for growth. Simulations were run with the reactions catalyzed by bcaT_1, bcaT_2 and bcaT_3 constrained to the catabolic direction.

A minimal medium was established by allowing uptake of glucose at a rate of 13.6 mmol gDW^-1 ^h^-1 ^and uptake of the previously determined essential amino acids at a rate of 0.5 mmol gDW^-1 ^h^-1 ^per amino acid. Remaining amino acids were individually supplied until growth was observed.

### Single gene/reaction deletion analyses

Single gene deletion (SGD) and single reaction deletion (SRD) analyses were performed using both FBA and MOMA. Reaction deletions were simulated by setting the corresponding flux to zero. Gene deletions were simulated by setting to zero all fluxes catalyzed by the corresponding gene product. Lethality was evaluated based on the deletions that led to infeasible problems or to biomass formation lower than 0.01 h^-1^. For simulation of growth on rich media, glucose uptake was set at 13.6 mmol gDW^-1 ^h^-1^, and all amino acids were allowed to be taken up (at a rate corresponding to the amino acid cell content assuming a specific growth rate of 0.8 h^-1^). Simulating growth on minimal media, only glucose at 13.6 mmol gDW^-1 ^h^-1^, arginine, methionine, valine and glutamante (0.5 mmol gDW^-1 ^h^-1^) were supplied.

### Modeling of homolactic and heterolactic metabolism

The shift from homolactic to heterolactic metabolism was simulated under anaerobic conditions, using FBA. Appropriated flux constraints were determined by fitting the model to experimental results, which is described in the following.

Two different approaches were selected to predict cell growth and product formation. First, glucose and amino acid uptake rates were set to fixed values and biomass production was maximized. Growth rate and products formation were determined as a function of different glucose uptake rates. Values for sugar uptake rates were taken from the literature [4,30], ranging from 7 to 24.6 mmol gDW^-1 ^h^-1^. Amino acids uptake rates were calculated from the amino acid cell content assuming a specific growth rate of 0.8 h^-1 ^[4]. A second approach consisted of setting a fixed specific growth rate while minimizing for the substrate uptake rates (objective function written as a linear combination of glucose and amino acids uptake rates, all terms with coefficient one). In this case, uptake rates and product formation were calculated for different specific growth rates. Here, a constraint on the flux of the pyruvate-formate lyase had to be introduced to account for experimental evidence, as suggested by Melchiorsen et al. (2002) [31].

### Design of a diacetyl overproducing mutant

Simulations were performed under aerobic conditions and on rich media (as described above), with glucose as the carbon source (13.6 mmol gDW^-1 ^h^-1^). Pyruvate secretion was not allowed and oxygen uptake was unconstrained. *In silico *gene deletions were simulated by constraining the respective fluxes to zero and solving the FBA problem while optimizing for biomass. Single gene deletions leading to high 2-acetolactate formation rates while allowing for growth (specific growth rate higher than 0.01 h^-1^) were selected as targets for metabolic engineering, as described in the Results and discussion section.

## List of abbreviations

LAB: Lactic Acid Bacteria

GRAS: Generally Recognized As Safe

FBA: Flux Balance Analysis

MOMA: Minimization Of Metabolic Adjustment

ORF: Open Reading Frame

PDH: Pyruvate dehydrogenase

PFL: Pyruvate-formate lyase

## Authors' contributions

APO carried out all aspects of the work and drafted the manuscript. JN conceived of the study and participated in its design and coordination. JF participated in the analysis of simulation results, conceived of the study, participated in its design and coordination and helped draft the manuscript. All authors read and approved the final manuscript.

## Supplementary Material

Additional File 1 List of reactions, ordered by metabolic pathway and alphabetically. List of reactions included in the metabolic network of *Lactococcus lactis*. Sheet "pathway_order" contains the reactions grouped by metabolic pathway, and journal references are indicated whenever reaction information is taken from journal references other than Bolotin et al. (2001). Sheet "alphabetic_order" lists the reactions alphabetically. Click here for file

Additional File 2 Biomass composition. Derivation of the equation for biomass composition, based on literature information. Click here for file

Additional File 3 Non-connected Metabolites. List of the non-connected metabolites and reactions. Click here for file

Additional File 4 Single gene and reaction deletions. List of genes and reactions from the metabolic network found to be lethal, ie, no growth was observed when setting the corresponding flux to zero and applying FBA or MOMA. Results are described both under anaerobic and aerobic conditions and both during growth on rich and minimal media. Click here for file

Additional File 5 Modeling the shift from homolactic ot heterolactic metabolism. These tables summarize the modeling procedure for inclusion of appropriate constrains when applying FBA to simulate anaerobic growth. Click here for file

## Figures and Tables

**Table 1:** Main characteristics of the reconstructed metabolic network of *Lactococcus lactis.*

**ORFs**	**358**
Experimental evidence	63
Clear function (functional annotation derived from probable homologues)	286
Tentative function (functional annotation derived from tentative homologues)	2
Putative function (sequence has probable homologues of uncertain function)	7
	
**Metabolites**	**509**
Intracellular metabolites	414
Extracellular metabolites	95
Unique metabolites	422
	
**Reactions**	**621**
Internal reactions	492
Exchange fluxes	129
	
Reactions with ORFs assignments	476
Reactions based on biochemical evidence / physiological considerations or inferred by the demands imposed on the metabolic reaction network	145

**Table 2:** Connectivity of metabolites in *Lactococcus lactis*. Connectivity is defined as the number of reactions in which the listed metabolites participate in.

**Metabolite**	**Connectivity**
ATP	117
proton	116
ADP	101
Phosphate	95
Pyruvate	60
Diphosphate	48
CO(2)	46
Glutamate	42
Phosphoenolpyruvate	39
NADP(+)	39
NAD(+)	38
NADPH	37
NADH	34
NH(3)	20

**Table 3:** Lethality of single gene deletions and single reaction deletions. Detailed data can be found in the Additional file 4.

Anaerobic	Rich media	FBA	# lethal reactions	71
			# lethal genes	33
		MOMA	# lethal reactions	81
			# lethal genes	38
	Minimal media	FBA	# lethal reactions	119
			# lethal genes	72
		MOMA	# lethal reactions	131
			# lethal genes	77
Aerobic	Rich media	FBA	# lethal reactions	72
			# lethal genes	34
		MOMA	# lethal reactions	84
			# lethal genes	41
	Minimal media	FBA	# lethal reactions	127
			# lethal genes	77
		MOMA	# lethal reactions	138
			# lethal genes	83

**Table 4:** Designed strategies to enhance 2-acetolactate production, and corresponding predictions for specific growth rates and yields of 2-acetolactate on glucose.

	**FBA**		**MOMA**	
	
	**Growth (h^-1^)**	**Y (C-mol/C-mol)**	**Growth (h^-1^)**	**Y (C-mol/C-mol)**
Reference	0.82	0.31	0.82	0.31
Δldh ΔaldB ΔadhA	0.82	0.32	0.82	0.31
Δldh ΔaldB ΔadhA Δpta	0.56	0.74	0.323	0.34
Δldh ΔaldB ΔadhA Δpta Δfhs	0.56	0.76	0.53	0.75
Δldh ΔaldB ΔadhA Δpta ΔserA	0.56	0.76	0.53	0.74
Δldh ΔaldB ΔadhA Δpta Δzwf	0.38	0.71	0.38	0.75

## References

[B1] Kleerebezem M, Hols P, Hugenholtz J (2000). Lactic acid bacteria as a cell factory: rerouting of carbon metabolism in Lactococcus lactis by metabolic engineering. Enzyme Microb Technol.

[B2] de Vos W (1996). Metabolic engineering of sugar catabolism in lactic acid bacteria. Antonie Van Leeuwenhoek.

[B3] Bolotin A, Wincker P, Mauger S, Jaillon O, Malarme K, Weissenbach J, Ehrlich SD, Sorokin A (2001). The complete genome sequence of the lactic acid bacterium Lactococcus lactis ssp. lactis IL1403. Genome Res.

[B4] Novak L, Loubiere P (2000). The metabolic network of Lactococcus lactis: distribution of (14)C- labeled substrates between catabolic and anabolic pathways. J Bacteriol.

[B5] van Niel EWJ, Hahn-Hagerdal B (1999). Nutrient requirements of lactococci in defined growth media. Applied Microbiology and Biotechnology.

[B6] Hugenholtz J, Kleerebezem M (1999). Metabolic engineering of lactic acid bacteria: overview of the approaches and results of pathway rerouting involved in food fermentations. Curr Opin Biotechnol.

[B7] Kleerebezem M, Boels IC, Groot MN, Mierau I, Sybesma W, Hugenholtz J (2002). Metabolic engineering of Lactococcus lactis: the impact of genomics and metabolic modelling. J Biotechnol.

[B8] Forster J, Famili I, Fu P, Palsson BO, Nielsen J (2003). Genome-scale reconstruction of the Saccharomyces cerevisiae metabolic network. Genome Res.

[B9] Edwards JS, Palsson BO (2000). Metabolic flux balance analysis and the in silico analysis of Escherichia coli K-12 gene deletions. BMC Bioinformatics.

[B10] Edwards JS, Palsson BO (1999). Systems properties of the Haemophilus influenzae Rd metabolic genotype. J Biol Chem.

[B11] Schilling CH, Covert MW, Famili I, Church GM, Edwards JS, Palsson BO (2002). Genome-scale metabolic model of Helicobacter pylori 26695. J Bacteriol.

[B12] Sheikh K, Forster J, Nielsen LK (2005). Modeling Hybridoma Cell Metabolism Using a Generic Genome-Scale Metabolic Model of Mus musculus. Biotechnol Prog.

[B13] Edwards JS, Ibarra RU, Palsson BO (2001). In silico predictions of Escherichia coli metabolic capabilities are consistent with experimental data. Nat Biotechnol.

[B14] Covert MW, Schilling CH, Famili I, Edwards JS, Goryanin II, Selkov E, Palsson BO (2001). Metabolic modeling of microbial strains in silico. Trends Biochem Sci.

[B15] Palsson B (2002). In silico biology through "omics". Nat Biotechnol.

[B16] Patil KR, Akesson M, Nielsen J (2004). Use of genome-scale microbial models for metabolic engineering. Curr Opin Biotechnol.

[B17] Segre D, Vitkup D, Church GM (2002). Analysis of optimality in natural and perturbed metabolic networks. Proc Natl Acad Sci U S A.

[B18] Andrade MA, Brown NP, Leroy C, Hoersch S, de Daruvar A, Reich C, Franchini A, Tamames J, Valencia A, Ouzounis C, Sander C (1999). Automated genome sequence analysis and annotation. Bioinformatics.

[B19] Benthin S (1992). Growth and product formation of Lactococcus cremoris.

[B20] Stephanopoulos G, Aristidou A, Nielsen J (1998). Metabolic Engineering: Principles and Methodologies.

[B21] Roissart H, Luquet FM, Roissart H and Luquet FM (1994). Bactéries Lactiques.

[B22] Jensen NB, Melchiorsen CR, Jokumsen KV, Villadsen J (2001). Metabolic behavior of Lactococcus lactis MG1363 in microaerobic continuous cultivation at a low dilution rate. Appl Environ Microbiol.

[B23] Nordkvist M, Jensen NBS, Villadsen J (2003). Glucose Metabolism in Lactococcus lactis MG1363 under Different Aeration Conditions: Requirement of Acetate To Sustain Growth under Microaerobic Conditions. Appl Environ Microbiol.

[B24] Henriksen CM, Nilsson D (2001). Redirection of pyruvate catabolism in Lactococcus lactis by selection of mutants with additional growth requirements. Appl Microbiol Biotechnol.

[B25] Pedersen MB, Koebmann BJ, Jensen PR, Nilsson D (2002). Increasing Acidification of Nonreplicating Lactococcus lactis{Delta}thyA Mutants by Incorporating ATPase Activity. Appl Environ Microbiol.

[B26] Neves AR, Ramos A, Shearman C, Gasson MJ, Santos H (2002). Catabolism of mannitol in Lactococcus lactis MG1363 and a mutant defective in lactate dehydrogenase. Microbiology.

[B27] Cocaign-Bousquet M, Garrigues C, Novak L, Lindley ND, Loubiere P (1995). Rational development of a simple synthetic medium for the sustained growth of Lactococcus lactis.. Journal of Applied Bacteriology.

[B28] Lai CY, Cronan JE (2003). Beta-ketoacyl-acyl carrier protein synthase III (FabH) is essential for bacterial fatty acid synthesis. J Biol Chem.

[B29] Kobayashi K, Ehrlich SD, Albertini A, Amati G, Andersen KK, Arnaud M, Asai K, Ashikaga S, Aymerich S, Bessieres P, Boland F, Brignell SC, Bron S, Bunai K, Chapuis J, Christiansen LC, Danchin A, Debarbouille M, Dervyn E, Deuerling E, Devine K, Devine SK, Dreesen O, Errington J, Fillinger S, Foster SJ, Fujita Y, Galizzi A, Gardan R, Eschevins C, Fukushima T, Haga K, Harwood CR, Hecker M, Hosoya D, Hullo MF, Kakeshita H, Karamata D, Kasahara Y, Kawamura F, Koga K, Koski P, Kuwana R, Imamura D, Ishimaru M, Ishikawa S, Ishio I, le Coq D, Masson A, Mauel C, Meima R, Mellado RP, Moir A, Moriya S, Nagakawa E, Nanamiya H, Nakai S, Nygaard P, Ogura M, Ohanan T, O'Reilly M, O'Rourke M, Pragai Z, Pooley HM, Rapoport G, Rawlins JP, Rivas LA, Rivolta C, Sadaie A, Sadaie Y, Sarvas M, Sato T, Saxild HH, Scanlan E, Schumann W, Seegers JFML, Sekiguchi J, Sekowska A, Seror SJ, Simon M, Stragier P, Studer R, Takamatsu H, Tanaka T, Takeuchi M, Thomaides HB, Vagner V, van Dijl JM, Watabe K, Wipat A, Yamamoto H, Yamamoto M, Yamamoto Y, Yamane K, Yata K, Yoshida K, Yoshikawa H, Zuber U, Ogasawara N (2003). Essential Bacillus subtilis genes. PNAS.

[B30] Thomas TD, Ellwood DC, Longyear VM (1979). Change from homo- to heterolactic fermentation by Streptococcus lactis resulting from glucose limitation in anaerobic chemostat cultures. J Bacteriol.

[B31] Melchiorsen CR, Jokumsen KV, Villadsen J, Israelsen H, Arnau J (2002). The level of pyruvate-formate lyase controls the shift from homolactic to mixed-acid product formation in Lactococcus lactis. Appl Microbiol Biotechnol.

[B32] Melchiorsen CR (2000). Metabolic Engineering of Pyruvate Metabolism in Lactococcus lactis.

[B33] Garrigues C, Loubiere P, Lindley ND, Cocaign-Bousquet M (1997). Control of the shift from homolactic acid to mixed-acid fermentation in Lactococcus lactis: predominant role of the NADH/NAD+ ratio. J Bacteriol.

[B34] Covert MW, Palsson BO (2002). Transcriptional regulation in constraints-based metabolic models of Escherichia coli. J Biol Chem.

[B35] Christensen JE, Dudley EG, Pederson JA, Steele JL (1999). Peptidases and amino acid catabolism in lactic acid bacteria. Antonie Van Leeuwenhoek.

[B36] Amarita F, Fernandez-Espla D, Requena T, Pelaez C (2001). Conversion of methionine to methional by Lactococcus lactis. FEMS Microbiol Lett.

[B37] Yvon M, Thirouin S, Rijnen L, Fromentier D, Gripon JC (1997). An aminotransferase from Lactococcus lactis initiates conversion of amino acids to cheese flavor compounds. Appl Environ Microbiol.

[B38] Jensen NB, Christensen B, Nielsen J, Villadsen J (2002). The simultaneous biosynthesis and uptake of amino acids by Lactococcus lactis studied by (13)C-labeling experiments. Biotechnol Bioeng.

[B39] Hugenholtz J, Kleerebezem M, Starrenburg M, Delcour J, de Vos W, Hols P (2000). Lactococcus lactis as a cell factory for high-level diacetyl production. Appl Environ Microbiol.

[B40] Lopez F, Starrenburg M, Hugenholtz J (1997). The role of NADH-oxidation in acetoin and diacetyl production from glucose in Lactococcus lactis subsp. lactis MG1363. FEMS Microbiology Letters.

[B41] Lopez F, Kleerebezem M, de Vos WM, Hugenholtz J (1998). Cofactor engineering: a novel approach to metabolic engineering in Lactococcus lactis by controlled expression of NADH oxidase. J Bacteriol.

[B42] Papp B, Pal C, Hurst LD (2004). Metabolic network analysis of the causes and evolution of enzyme dispensability in yeast. Nature.

[B43] Akesson M, Forster J, Nielsen J (2004). Integration of gene expression data into genome-scale metabolic models. Metab Eng.

[B44] Covert MW, Knight EM, Reed JL, Herrgard MJ, Palsson BO (2004). Integrating high-throughput and computational data elucidates bacterial networks. Nature.

[B45] Patil KR, Nielsen J (2005). Uncovering transcriptional regulation of metabolism by using metabolic network topology. Proc Natl Acad Sci U S A.

[B46] Snoep JL, de Graef MR, Westphal AH, de KA, Teixeira de Mattos MJ, Neijssel OM (1993). Differences in sensitivity to NADH of purified pyruvate dehydrogenase complexes of Enterococcus faecalis, Lactococcus lactis, Azotobacter vinelandii and Escherichia coli: implications for their activity in vivo. FEMS Microbiol Lett.

[B47] Schlegel HG (1992). Allgemeine Mikrobiologie (7th Ed).

