# Supplementary material for: Modeling Lactococcus lactis using a genome-scale flux model
Source: BMC Microbiol. 2005 Jun 27;5:39. doi: 10.1186/1471-2180-5-39 (PMC1185544; doi:10.1186/1471-2180-5-39)
Supplement: Additional File 2 — Biomass composition. Derivation of the equation for biomass composition, based on literature information. [file 1471-2180-5-39-S2.pdf]

BIOMASS COMPOSITION

B.1 Overall cellular composition of *Lactococcus lactis*

The macromolecular composition of bacterial cells is dependent on the growth conditions (growth rate and limiting substrate). For example, as the growth rate increases the cellular content of RNA usually increases, while the protein and DNA content decreases (Novak, L. *et al.*, 2000). The relative content of cell wall polymers also decreases with the increase of the growth rate (Benthin, S., 1992).

However, for simplicity, a biomass equation was defined as follows. A average macromolecular composition was set, based on data available from the literature. Biomass was subdivided into seven components - protein, RNA, DNA, lipids, lipoteichoic acid, peptidoglycan and polysaccharides - accounting for the cell dry weight (see Table B.1). Inorganic ions and free metabolites were negligible for modelling purposes.

B.2 Protein composition

Protein fraction is meant to account for the cellular proteins. The amino acid composition of the protein fraction of *L. lactis* was approximated by the data for *L. lactis* NCDO 2118 (Novak, L. *et al.*, 2000). The composition has been corrected for peptidoglycan amino acids since the original data was derived from total cell hydrolysates (see Table B.2). An average molecular weight for the protein fraction was calculated from the molecular weight of the amino acid residues (as polymerisation is usually associated with the loss of one H_2O molecule).

Table B.1 - Overall cellular composition of *Lactococcus. lactis* (mass percent).

	Overall cellular composition of *L. lactis*				
(% w/w)	Novák and Loubiere[1]	Benthin[2]	Chong[3]	Modified Novák and Loubiere[4]	Average cellular composition[5]
**Protein**	45,0	45,0	54,0	39,1	**46,0**
**DNA**	3,0	2,0	2,0	3,0	**2,3**
**RNA**	8,0	12,0	12,0	8,0	**10,7**
**Lipoteichoic acids**	10,0	8,0	6,0	10,0	**8,0**
**Lipids**	4,3	3,0	3,0	4,3	**3,4**
**Peptidoglycan**	-	12,0	12,0	11,4	**11,8**
Amino sugars	5,5	-	-	-	-
**Polysaccharides**	12,0	-	-	12,0	**12,0**
Wall polysaccharides	-	-	2,0	-	-
Inorganic ions	7,0	5,0	1,0	7,0	4,3
Free aminoacids	-	5,0	4,0	-	4,5
Free nucleotides	-	3,0	3,0	-	3,0
Metabolic intermediates	-	2,0,	1,0	-	1,5

[1] Average composition of *L. lactis* subsp *lactis* NCDO 2118, during anaerobic growth on MCD medium, batch conditions (Novak, L. *et al.*, 2000). The protein fraction represents the cellular proteins and the cell wall peptides.
[2] Average composition of lactococcal cells at intermediate growth rates. Conditions not specified. (Benthin, S., 1992)
[3] Average composition of *L. lactis* subsp *cremoris.* Conditions not specified. (Chong, B. F., 2002)
[4] Some as [1] but accounting for peptidoglycan (PG). From information on the PG composition (see below), the percentage of PG in the cell was calculated from the amino sugars, assuming that all amino sugars are part of the PG fraction: $\%(\mathrm{PG}) = \frac{\mathrm{MW(PG)}}{\mathrm{MW(aminosugars)}} \times \%(\mathrm{aminosugars})$. Aminoacids in the PG fraction were discounted from the protein fraction.
[5] Average of [2], [3], and [4]. Only the fractions marked as bold were considered to account for the cellular dry weight.

Table B.2 – Amino acid composition of the Protein fraction in *Lactococcus lactis*.

	**Protein composition**		
	Novák and Loubiere[1]	Average aminoacid content in the Protein fraction[2]	MW of residue
	% (mol a.a./mol proteins)	%(mol a.a./mol Protein)	(g/mol)
Alanine	12,6	8,6	71,08
Arginine	3,6	4,1	156,19
Aspartate (Asx)	(10,2)	3,1	114,10
Asparagine		5,9	115,09
Cysteine	3,0	3,4	103,14
Glutamate (Glx)	(11,1)	3,6	128,13
Glutamine		6,4	129,12
Glycine	8,1	9,2	57,05
Histidine	1,4	1,5	137,14
Isoleucine	5,4	6,1	113,16
Leucine	7,6	8,7	113,16
Lysine	8,8	7,2	128,17
Methionine	2,2	2,5	131,19
Phenylalanine	3,3	3,8	147,18
Proline	3.1	3,5	97,12
Serine	4,5	5,1	87,08
Threonine	5,0	5,6	101,10
Tryptophan	1,5	1,7	186,21
Tyrosine	2,4	2,7	163,18
Valine	6,4	7,2	99,13
		**Average Molecular Weight of Protein**	**109,6**

[1] Average amino acid composition of proteins (cellular proteins and cell wall peptides) of *L. Lactis* subsp. *lactis* NCDO, during anaerobic growth on MCD and MS14 media, batch conditions (Novak, L. *et al.*, 2000). Asx = sum of aspartate and asparagine. Glx = sum of glutamate and glutamine.

[2] Amino acid composition in the Protein fraction (PROT), corrected for peptidoglycan amino acids through an interactive mass balance: $m_{aa,\,PROT} = m_{aa,proteins} - m_{aa,\,PG}$, where,

$$m_{aa,proteins} = \frac{\text{mol a.a. in proteins}}{\text{mol proteins}} \times \frac{\times\,\text{MW(residue)}}{\text{MW(proteins)}} \times \frac{\text{g proteins}}{\text{g DW}}$$

$$m_{aa,PG} = \frac{\text{mol a.a. in PG}}{\text{mol PG}} \times \frac{\text{MW(residue)}}{\text{MW(PG)}} \times \frac{\text{g PG}}{\text{g DW}} \quad \text{and}$$

$$m_{aa,PROT} = \frac{\text{mol a.a. in PROT}}{\text{mol PROT}} \times \frac{\text{MW(residue)}}{\text{MW(PROT)}} \times \frac{\text{g PROT}}{\text{g DW}}\ .$$

Asx (Glx) was simply divided into two halves, “Asp + Asn” (“Glu + Gln”), before mass balances had been applied.

B.3 Nucleotide composition

The composition considered for DNA and RNA are presented in Tables B.3 and B.4, respectively. Average molecular weights for were calculated from the molecular weight of the nucleotide residues.

Table B.3 – Deoxyribonucleotide composition of the DNA fraction in *Lactococcus lactis*.

	**DNA composition**	
	% (mol/mol)[1]	MW of residue (g/mol)
dAMP	32,3	331,2
dTMP	32,3	304,2
dCMP	17,7	289,2
dGMP	17,7	304,2
**Average Molecular Weight of DNA**		**314,7**

[1] The deoxyribonucleotide composition was based on the GC content of 35,4% determined by Bolotin, A. *et al.* (2001) for *L. lactis* subsp *lactis* IL 1403.

Table B.4 - Ribonucleotide composition of the RNA fraction in *Lactococcus lactis*.

	**RNA composition**	
	% (mol/mol)[1]	MW of residue (g/mol)
AMP	26,2	329,2
UMP	21,6	306,2
CMP	20,0	305,2
GMP	32,2	345,2
**Average Molecular Weight of RNA**		**324,6**

[1] The ribonucleotide composition was based on values for *Escherichia coli* (Neidhardt, F. C. *et al.*, 1987)

B.4 Lipids composition

The lipid fraction was considered to be composed by five different types of phospholipids (see Table B.5). An average fatty acid composition was taken into account for calculations (Table B.6).

Table B.5 – Lipid composition in *Lactococcus lactis*.

	Lipid composition	
	% (mol/mol)[1]	MW (g/mol)[2]
Phosphatidylglycerol	18,9	754,3
Cardiolipin	42,5	1413,6
Lysophosphatidylglycerol	4,3	500,2
Diglucosyl diacylgycerol	30,3	924,3
Monoglucosyl diacylgycerol	4,0	762,3
**Average Molecular Weight of Lipids**		**1075,4**

[1] Lipid composition was approximated to the membrane lipid composition of *L. lactis* subsp *cremoris* (Driessen, A. J. *et al.*, 1988).
[2] Molecular weight was calculated from the elementary composition. To account for the acyl molecular weight, an average MW of fatty acids was considered: MW(fatty acids without a –COOH group) = 227,15 g/mol.

Table B.6 – Lipid fatty acid composition of *Lactococcus lactis*.

	Fatty Acid composition			
	%(w/w)[1]	%(w/w) (w/o Others)[2]	%(mol/mol) (w/o Others)	MW (g/mol)
Myristic acid (14:0)	7,6	7,7	9,13	228,4
Tetradecenoic acid (14:1)	0,5	0,5	0,61	226,4
Hexadecanoic acid (16:0)	27,5	27,9	29,6	256,4
Hexadecenoic acid (16:1)	2,5	2,5	2,7	254,4
Octadecanoic acid (18:0)	1,2	1,2	1,2	284,5
Octadecenoic acid (18:1)	44,9	45,5	43,8	282,5
11,12-methylene-octadecanoic acid (Δ19:0)	14,5	14,7	13,5	296,5
Others	1,3	-	-	-
**Average Molecular Weight of Fatty Acid**				**272,15**

[1] Average mass composition of the fatty acid fraction in *L. lactis* subsp. *cremoris* NCDO763, growing in a chemical defined medium at 30ºC (Guillot, A. *et al.*, 2000)
[2] Same as [1] but not considering the undetermined fraction of “Others”. Known fraction was recalculated to account for 100%.

B.5 Lipoteichoic acid composition

The structural unit of the lipoteichoic acid was considered to be a linear polymer of n=16 glycerophosphate residues covalently linked to a lipid moiety of diglucosyl diacylglycerol, that serves as anchor to the membrane. 61 % of the residues are substituted with D-galactose and 38% are substituted with L-alanine (see Table B.7).

Table B.7 – Lipoteichoic acid composition in *Lactococcus lactis*.

	LTA composition	
	Average molar ratio[1]	MW of residue (g/mol)
Glycerol phosphate	16	154,0
L-alanine	6,08	71,1
D-galactose	9,76	162,1
Diglucosyl diacylglycerol	1	906,3
**Average Molecular Weight of LTA**		**5384,7**

[1] Glycerol phosphate, alanine and galactose were considered to be present in the ratio 1:0,38:0,61 in each molecule (Schurek, J. *et al.*, 1989). Each molecule was considered to have 16 glycerophosphate residues, linked to one anchor of diglucosyl diacylglycerol (Buist, G., 1997).

B.6 Peptidoglycan composition

The peptidoglycan fraction is formed by a backbone of amino sugars with a pentapeptide tail linked to the murein molecule. All building blocks were considered to be present in the same molar ratio (Table B.8).

Table B.8 – Peptidoglycan composition in *Lactococcus lactis*.

	**PG composition**	
	Average molar ratio[3]	MW of residue (g/mol)
Amino sugars[1]		
N-acetylmuramate	1	275,3
N-acetyl-D-glucosamine	1	203,2
Amino acids[2]		
L-alanine	1	142,2
D-glutamate	1	128,1
L-lysine	1	128,2
D-alanine	1	142,2
D-aspartate	1	114,1
	**Average Molecular Weight of PG**	**991,0**

[1] Amino sugars composition from Delcour, J. *et al* (1999)
[2] Amino acids composition from de Ambrosini, V. *et al* (1996) and Bolotin, A. *et al* (2001)
[3] N-acetylmuramate and N-acetyl-D-glucosamine are arranged alternately to form the backbone of the peptidoglycan molecule. N-acetylmuramate molecules are cross-linked with a tail of four aminoacids (L-Ala, D-Glu, L-Lys, D-Ala). Some of those tails are linked together through an interbridge of D-aspartate. According to de Ambrosini, V. *et al* (1996), D-Asp is present is the same molar proportion as L-Lys and D-Glu.

B.7 Polysaccharide composition

The structural unit for the polysaccharide fraction was set based on 1 mole of galactose. Polysaccharide composition is presented in Table B.9.

Table B.9 – Polysaccharides composition in *Lactococcus lactis*.

	POLYS composition		
	Average mass ratio[1]	Average molar ratio	MW of residue (g/mol)
D-glucose	5,5	5,5	162
D-rhamnose	5,1	5,6	145
D-galactose	1,0	1,0	162
**Average Molecular Weight of POLYS**			**1872,9**

[1] Polysaccharide mass fraction observed for *L. lactis* subsp. *lactis* NCDO, during anaerobic growth on MCD medium, batch conditions (Novak, L. *et al.*, 2000).

B.8 Energy requirement for polymerisation of macromolecules

The energy requirements for polymerisation of macromolecules (Table B.10) have been approximated by the values for *E. coli* (Neidhardt, F. C. *et al.*, 1987)

Table B.10 – Energy requirements for the polymerization of macromolecules.

**Process**	**Energy required**[1]
Protein synthesis and processing	(μmol ATP / μmol aminoacid)
Activation and incorporation	4,0
mRNA synthesis	0,2
Proofreading	0,1
Assembly and modification	0,006
RNA synthesis and processing	(μmol ATP / μmol RNA)
Discarding segments	0,38
Modification	0,02
DNA synthesis and processing	(μmol ATP / μmol DNA)
Unwinding helix	1,0
Proofreading	0,36
Discontinuous synthesis	0,006
Negative supercoiling	0,005
Methylation	0,001

[1] Data for *Escherichia coli* (Neidhardt, F. C. *et al.*, 1987)

B.8.1 Protein assembly

From the amino acid composition (Table B.2) and energy requirements (Table B.10) for the polymerisation of the protein fraction, the protein assembly equation, PROTass, can be written as

8,6 L-alanine + 4,1 L-arginine + 3,1 L-aspartate + 5,9 L-asparagine + 3,4 L-cysteine + 3,6 L-glutamate + 6,4 L-glutamine + 9,2 glycine + 1,5 L-histidine + 6,1 L-isoleucine + 8,7 L-leucine + 7,2 L-lysine + 2,5 L-methionine + 3,8 L-phenylalanine + 3,5 L-proline + 5,1 L-serine + 5,6 L-threonine + 1,7 L-tryptophan + 2,7 L-tyrosine + 7,2 L-valine + 430,6 ATP -> 100 PROT + 430,6 ADP + 430,6 phosphate

B.8.2 DNA assembly

DNA assembly reaction can be simply described as DNA_n + dNMP→ DNA_{n+1}, where dNMP represents a deoxyribonucleotide monophosphate. However, as DNA synthesis takes deoxyribonucleotide triphosphates as precursors, DNA assembly costs an additional 2 mol ATP per mol DNA. From Table B.3 and Table B.10, DNA assembly equation, DNAass, is defined as

32,3 dAMP + 17,7 dCMP + 17,7 dGMP + 32,3 dTMP + 337,2 ATP -> 100 DNA + 337,2 ADP + 337,2 phosphate

B.8.3 RNA assembly

As for DNA, RNA assembly reaction can be described as RNA_n + NMP→ RNA_{n+1}, NMP being a ribonucleotide monophosphate. However, as RNA synthesis takes ribonucleotide triphosphates as precursors, RNA assembly costs an additional 2 mol ATP per mol RNA. From Table B.4 and Table B.10, RNA assembly equation, RNAass, is defined as

26,2 AMP + 20 CMP + 32,2 GMP + 21,6 UMP + 240 ATP -> 100 RNA + 240 ADP + 240 phosphate

B.9 Biomass assembly

Biomass assembly reaction can be written as the sum of all macromolecule fractions. Macromolecular composition of *L. lactis* is summarized in Table B.11

Table B.11 – Overall macromolecular composition of *Lactococcus lactis*.

	**Macromolecular composition of *L. lactis***			
Macromolecule	% (w/w)	% (mol/mol)	mmol/gDW	$MW_{macromolecule}$ (g/mol)
Protein	46,0	86,9	4,201	109,6
DNA	2,3	1,5	0,074	314,7
RNA	10,	6,8	0,329	324,6
LTA	8,0	0,3	0,015	5384,7
Lipid	3,4	0,7	0,032	1075,4
Peptidoglycan	11,8	2,5	0,119	991,0
Polysaccharide	12,0	1,3	0,064	1871,9

Furthermore, the growth associated ATP for maintenance was estimated to be 18.15 mmol / gDW. This value was found by fitting the reconstructed model to a expected growth rate of 0,8 h^{-1} (Novak, L. *et al.*, 2000), when setting glucose uptake to 13,6 mmol/gDW.h), all amino acids to a fixed uptake rate (calculated from the amino acid cell content, at a dilution rate of 0,8 h^{-1}) and maximizing for biomass. However, this value possesses high uncertainty and may need to be readjusted.

Considering a basis of 1 gDW, the biomass assembly equation, BIOMass, is defined as

4,201 PROT + 0,074 DNA + 0,329 RNA + 0,015 LTA + 0,032 LIP + 0,119 PG + 0,064 POLYS + 18.15 ATP -> BIOMASS + 18.15 ADP + 18.15 phosphate

B.10 Elemental composition

An elemental composition for the *in silico* reconstructed strain was calculated from an elemental balance over each component of the biomass. Elemental composition of each of the biomass components is provided in Table B.12.

Table B.12 – Elemental composition of each of the components of the biomass.

	C	H	N	O	P	S		C	H	N	O	P	S
**Proteins**							**Fatty acids**						
Alanine	3	7	1	2	0	0	C14:0	13	27	0	0	0	0
Arginine	6	14	4	2	0	0	C14:1	13	25	0	0	0	0
Asparagine	4	8	2	3	0	0	C16:0	15	31	0	0	0	0
Aspartate	4	7	1	4	0	0	C16:1	15	29	0	0	0	0
Cysteine	3	7	1	2	0	1	C18:0	17	35	0	0	0	0
Glutamate	5	9	1	4	0	0	C18:1	17	33	0	0	0	0
Glutamine	5	10	2	3	0	0	ΔC19:0	18	35	0	0	0	0
Glycine	2	5	1	2	0	0	**Lipids**						
Histidine	6	9	3	2	0	0	phosphatidylglycerol	40,2	77	0	10	1	0
Isoleucine	6	13	1	2	0	0	cardiolipin	78,4	147	0	16	2	0
Leucine	6	13	1	2	0	0	lysophosphatidylgly.	23,1	46	0	9	1	0
Lysine	6	14	2	2	0	0	di. diacylglycerol	49,2	90	0	15	0	0
Methionine	5	11	1	2	0	1	mono. diacylglycerol	43,2	80	0	10	0	0
Phenylalanine	9	11	1	2	0	0	**Peptidoglycan**						
Proline	5	9	1	2	0	0	N-Acetylmuramate	11	19	1	8	0	0
Serine	3	7	1	3	0	0	N-acetyl-D-glucosamine	8	15	1	6	0	0
Threonine	4	9	1	3	0	0	Alanine	3	7	1	2	0	0
Tryptophan	11	12	2	2	0	0	Glutamate	5	9	1	4	0	0
Tyrosine	9	11	1	3	0	0	Lysine	6	14	2	2	0	0
Valine	5	11	1	2	0	0	Aspartate	4	7	1	4	0	0
**DNA**							**Lipoteichoic acid**						
dAMP	10	16	5	7	1	0	glycerol phosphate	3	9	0	6	1	0
dCMP	9	14	3	7	1	0	alanine	3	7	1	2	0	0
dGMP	10	14	5	7	1	0	galactose	6	12	0	6	0	0
dTMP	10	15	2	8	1	0	diglu. diacylglycerol	49,2	90	0	15	0	0
**RNA**							**Polysaccharides**						
AMP	10	14	5	7	1	0	glucose	6	12	0	6	0	0
CMP	9	14	3	8	1	0	rhamnose	6	12	0	5	0	0
GMP	10	14	5	8	1	0	galactose	6	12	0	6	0	0
UMP	9	13	2	9	1	0							

Considering the percentage contribution of each of the components to the overall cell, an approximate elemental biomass composition was determined: $CH_{1,95}O_{0,63}N_{0,22}P_{0,02}S_{0,01}$. Therefore, the molecular weight for the reconstructed *L. lactis* on a C-mole basis is 27,8 g/C-mol.
