# Supplementary material for: Modeling Lactococcus lactis using a genome-scale flux model
Source: BMC Microbiol. 2005 Jun 27;5:39. doi: 10.1186/1471-2180-5-39 (PMC1185544; doi:10.1186/1471-2180-5-39)
Supplement: Additional File 3 — Non-connected Metabolites. List of the non-connected metabolites and reactions. [file 1471-2180-5-39-S3.pdf]

Table 7 – List of the 28 intracellular metabolites not connected into the overall metabolic network. These non-connected metabolites take part in 25 "non-connected" reactions, catalyzed by 21 "non-connected" gene products.

**Metabolite name**	**Reaction name**	**ORF**	**Reaction**
(R)-pantoate	panE_1	panE	(R)-pantoate + NADP(+) <-> 2-dehydropantoate + NADPH
1,4-beta-D-xylan	xynB_1	xynB	1,4-beta-D-xylan + H(2)O <-> 2 D-xylose
2-deoxy-D-ribose 1-phosphate	deoB_1	deoB	2-deoxy-D-ribose 1-phosphate <-> 2-deoxy-D-ribose 5-phosphate
2-oxo-3-hexenodiote	xylH_1	xylH	2-oxo-4-hexenediote <-> 2-oxo-3-hexenodiote
2-oxo-4-hexenediote	xylH_1	xylH	2-oxo-4-hexenediote <-> 2-oxo-3-hexenodiote
4-carboxymuconolactone	pcaC_1	pcaC	4-carboxymuconolactone -> 5-oxo-4,5-dihydrofuran-2-acetate + CO(2)
4-methyl-5-(2-hydroxyethyl)-thiazole	thiM_1	thiM	ATP + 4-methyl-5-(2-hydroxyethyl)-thiazole -> ADP + 4-methyl-5-(2- phosphoethyl)-thiazole
5-oxo-4,5-dihydrofuran-2-acetate	pcaC_1	pcaC	4-carboxymuconolactone -> 5-oxo-4,5-dihydrofuran-2-acetate + CO(2)
6-phospho-beta-D-glucoside-(1,4)-D-glucose	bglA_1	bglA	6-phospho-beta-D-glucoside-(1,4)-D-glucose + H(2)O -> D-glucose 6-phosphate + D-glucose
beta-D-galactose	galM_1	galM	D-galactose <-> beta-D-galactose
beta-D-xylose	xylM_2	xylM	D-xylose <-> beta-D-xylose
beta-lactose	xylM_4	xylM	lactose <-> beta-lactose
beta-L-arabinose	xylM_3	xylM	L-arabinose <-> beta-L-arabinose
beta-maltose	xylM_5	xylM	maltose <-> beta-maltose
cellobiose	bglS_2	bglS	cellobiose + H(2)O -> 2 beta-D-glucose
D-galacturonate	uxaC_2	uxaC	D-galacturonate <-> D-tagaturonate
D-glucuronate	uxaC_1	uxaC	D-glucuronate <-> D-fructuronate
gentobiose	bglS_1	bglS	gentobiose + H(2)O -> 2 beta-D-glucose
glycolaldehyde	folB_1	folB	2-amino-4-hydroxy-6-(erythro-1,2,3-trihydroxypropyl)dihydropteridine -> 2-amino-4-hydroxy-6-hydroxymethyl-7,8-dihydropteridine + glycolaldehyde
indol	trpB_1	trpB	L-serine + indol <-> L-tryptophan + H(2)O
indol-3-carboxaldehyde	pI3CA_1		indol-3-carboxaldehyde -> I3CAext
L-arabinose	xylM_3	xylM	L-arabinose <-> beta-L-arabinose
menaquinone	unk_48		menaquinol <-> menaquinone + 2 H(+)
N-acetyl-D-glucosamine 6-phosphate	nagA_1	nagA	N-acetyl-D-glucosamine 6-phosphate + H(2)O <-> D-glucosamine 6-phosphate + acetate
superoxide	sodA_1	sodA	2 superoxide + 2 H(+) -> O(2) + H(2)O(2)
thymine	pdp_3	pdp	thymidine + phosphate <-> thymine + D-ribose 1-phosphate
undecaprenol	bacA_1	bacA	ATP + undecaprenol <-> ADP + undecaprenyl phosphate
xylobiose	xynB_2	xynB	xylobiose + H(2)O <-> 2 D-xylose
