# Supplementary material for: Modeling Lactococcus lactis using a genome-scale flux model
Source: BMC Microbiol. 2005 Jun 27;5:39. doi: 10.1186/1471-2180-5-39 (PMC1185544; doi:10.1186/1471-2180-5-39)
Supplement: Additional File 4 — Single gene and reaction deletions. List of genes and reactions from the metabolic network found to be lethal, ie, no growth was observed when setting the corresponding flux to zero and applying FBA or MOMA. Results are described both under anaerobic and aerobic conditions and both during growth on rich and minimal media. [file 1471-2180-5-39-S4.pdf]

SINGLE GENE & REACTION DELETIONS

D.1 Lethal Genes

Underlined genes were found in the literature as being lethal during growth in rich media.

Simple underline: gene genes reported to be lethal for *Bacillus subtilis* (Kobayashi, K. *et al.*, 2003).

Double underline: reported in Lai, C.Y. *et al.* (2003).

Dot underline: Karin Hammer, personal communication.

Anaerobic, Rich Media

FBA: *ADK CDSA CPSM DAL DDL DFRA DGKA FABD FABH FBAA FEMD GLMU GPDA HASC MRAY MURB MURC MURD MURE MURF MURG MURI PFK PGK PGSA PSTC PYRH RACD RMLA RMLB RMLC RPIA THYA*

ALAextO ARGextI BIOMass bPOLYS c140s c141s c160s c161s c180s c181s CYSextO d190s DIPHOS DNAass HISextO LIPass lump LYSextO METextI pMET PROextO PROTass RNAass TDPk UDPk unk

MOMA: *ADHA ADHE ADK CDSA CPSM DAL DDL DFRA DGKA FABD FABH FBAA FEMD GLMU GPDA HASC MRAY MURB MURC MURD MURE MURF MURG MURI PFK PFL PGIA PGK PGSA PSTC PYRH RACD RMLA RMLB RMLC RPIA THYA TPIA*

ALAextO ARGextI BIOMass bPOLYS c140s c141s c160s c161s c180s c181s CYSextO d190s DIPHOS DNAass ETOHextO FORMextO GLUCextI HISextO LIPass lump LYSextO METextI pETOH pFORM pMET PROextO PROTass RNAass TDPk UDPk unk

Anaerobic, Minimal Media

FBA: *ADK AROA AROB AROC AROD AROE AROK CDSA CPSM CYSE DAL DAPA DAPB DDL DFRA DGKA FABD FABH FBAA FEMD GLMU GPDA GUAA GUAB HASC HISA HISB HISC HISD HISG HISH HISI HISK ILVB ILVC ILVD LEUA LEUB LEUC LEUD LYSA MRAY MURB MURC MURD MURE MURF MURG MURI PEPV PFK PFL PGK PGSA PHEA PROA PROB PROC PSTC PURH PYCA PYRH RACD RMLA RMLB RMLC RPIA THYA TKT YAFB YCHH YWIC*

ARGextI BIOMass bPOLYS c140s c141s c160s c161s c180s c181s chem d190s DIPHOS DNAass GDPk GLUCextI GLUextI LIPass lump METextI pMET PROTass RNAass SLFextI TDPk UDPk unk

MOMA: *ADHA ADHE ADK AROA AROB AROC AROD AROE AROK CDSA CPSM CYSE DAL DAPA DAPB DDL DFRA DGKA FABD FABH FBAA FEMD GLMU GPDA GUAA GUAB HASC HISA HISB HISC HISD HISG HISH HISI HISK ILVB ILVC ILVD LEUA LEUB LEUC LEUD LYSA MRAY MURB MURC MURD MURE MURF MURG MURI PEPV PFK PFL PGIA PGK PGSA PHEA PROA PROB PROC PSTC PURH PYCA PYRH RACD RMLA RMLB RMLC RPIA THYA TKT TPIA YAFB YCHH YWIC ZWF*

ARGextI BIOMass bPOLYS c140s c141s c160s c161s c180s c181s chem CO2extO d190s DIPHOS DNAass ETOHextO FORMextO GDPk GLUCextI GLUextI LIPass lump METextI pCO2 pETOH pFORM pMET PROTass RNAass SLFextI TDPk UDPk unk

Aerobic, Rich Media

FBA: *ADK CDSA CPSM DAL DDL DFRA DGKA FABD FABH FBAA FEMD GLMU GLYA GPDA HASC MRAY MURB MURC MURD MURE MURF MURG MURI PFK PGK PGSA PSTC PYRH RACD RMLA RMLB RMLC RPIA THYA*

ALAextO ARGextI BIOMass bPOLYS c140s c141s c160s c161s c180s c181s CYSextO d190s DIPHOS DNAass HISextO LIPass lump LYSextO METextI pMET PROextO PROTass RNAass TDPk UDPk unk

MOMA: *ADHA ADHE ADK CDSA CPSM DAL DDL DFRA DGKA FABD FABH FBAA FEMD GLMU GLYA GPDA HASC MRAY MURB MURC MURD MURE MURF MURG MURI PDHC PDHD PFK PGIA PGK PGSA PSTC PYK PYRH RACD RMLA RMLB RMLC RPIA THYA TPIA*

ALAextO ARGextI BIOMass bPOLYS c140s c141s c160s c161s c180s c181s CO2extO CYSextO d190s DIPHOS DNAass ETOHextO GLUCextI HISextO LIPass lump LYSextO METextI pCO2 pETOH pMET PROextO PROTass RNAass TDPk UDPk unk

Aerobic, Minimal Media

FBA: *ADK AROA AROB AROC AROD AROE AROK CDSA CPSM CYSE DAL DAPA DAPB DDL DFRA DGKA FABD FABH FBAA FEMD FOLD GLMU GLYA GPDA GUAA GUAB HASC HISA HISB HISC HISD HISG HISH HISI HISK ILVB ILVC ILVD LEUA LEUB LEUC LEUD LYSA MRAY MURB MURC MURD MURE MURF MURG MURI PEPV PFK PGK PGSA PHEA PROA PROB PROC PSTC PURH PYCA PYRH RACD RMLA RMLB RMLC RPIA SERA SERB SERC THYA TKT TPIA YAFB YCHH YWIC*

ARGextI BIOMass bPOLYS c140s c141s c160s c161s c180s c181s d190s chem. DIPHOS DNAass GDPk GLUCextI GLUextI LIPass lump METextI O2extI pMET pO2 PROTass RNAass SLFextI TDPk UDPk unk

MOMA: *ADHA ADHE ADK AROA AROB AROC AROD AROE AROK CDSA CPSM CYSE DAL DAPA DAPB DDL DFRA DGKA FABD FABH FBAA FEMD FOLD GLMU GLYA GPDA GUAA GUAB HASC HISA HISB HISC HISD HISG HISH HISI HISK ILVB ILVC ILVD LEUA LEUB LEUC LEUD LYSA MRAY MURB MURC MURD MURE MURF MURG MURI PDHC PDHD PEPV PFK PGIA PGK PGSA PHEA PROA PROB PROC PSTC PURH PYCA PYRH RACD RMLA RMLB RMLC RPIA SERA SERB SERC THYA TKT TPIA YAFB YCHH YWIC ZWF*

ARGextI BIOMass bPOLYS c140s c141s c160s c161s c180s c181s chem CO2extO d190s DIPHOS DNAass ETOHextO GDPk GLUCextI GLUextI LIPass lump METextI O2extI pCO2 pETOH pMET pO2 PROTass RNAass SLFextI TDPk UDPk unk

D.2 Lethal Reactions

<u>Anaerobic, Rich Media</u>

FBA: adk_1 ALAextO ARGextI BIOMass bPOLYS c140s_1 c141s_1 c160s_1 c161s_1 c180s_1 c181s_1 cdsA_1 cdsA_2 cpsM_1 CYSextO d190s_1 dal_1 ddl_1 dfrA_1 dgkA_1 DIPHOS DNAass fabD_1 fabH_1 fbaA_1 femD_1 femD_2 glmU_1 glmU_2 gpdA_1 hasC_1 HISextO LIPass lump_1 lump_2 lump_3 lump_4 LYSextO METextI mraY_1 murB_1 murC_1 murD_1 murE_1 murF_1 murG_1 murI_1 pfk_1 pgk_1 pgsA_1 pgsA_2 pMET_1 PROextO PROTass pyrH_1 racD_1 rmlA_1 rmlB_1 rmlC_1 RNAass rpiA_1 TDPk_1 thyA_1 UDPk_1 unk_42 unk_43 unk_44 unk_45a unk_45b unk_46 unk_47

MOMA: adhA_1 adhE_1 adk_1 ALAextO ARGextI BIOMass bPOLYS c140s_1 c141s_1 c160s_1 c161s_1 c180s_1 c181s_1 cdsA_1 cdsA_2 cpsM_1 CYSextO d190s_1 dal_1 ddl_1 dfrA_1 dgkA_1 DIPHOS DNAass ETOHextO fabD_1 fabH_1 fbaA_1 femD_1 femD_2 FORMextO glmU_1 glmU_2 GLUCextI gpdA_1 hasC_1 HISextO LIPass lump_1 lump_2 lump_3 lump_4 LYSextO METextI mraY_1 murB_1 murC_1 murD_1 murE_1 murF_1 murG_1 murI_1 pETOH_1 pfk_1 pfl_1 pFORM_1 pgiA_1 pgk_1 pgsA_1 pgsA_2 pMET_1 PROextO PROTass pyrH_1 racD_1 rmlA_1 rmlB_1 rmlC_1 RNAass rpiA_1 TDPk_1 thyA_1 tpiA_1 UDPk_1 unk_42 unk_43 unk_44 unk_45a unk_45b unk_46 unk_47

<u>Anaerobic, Rich Media</u>

FBA: adk_1 ARGextI aroA_1 aroB_1 aroC_1 aroD_1 aroE_1 aroK_1 BIOMass bPOLYS c140s_1 c141s_1 c160s_1 c161s_1 c180s_1 c181s_1 cdsA_1 cdsA_2 chem_2 cpsM_1 cysE_1 d190s_1 dal_1 dapA_1 dapB_1 ddl_1 dfrA_1 dgkA_1 DIPHOS DNAass fabD_1 fabH_1 fbaA_1 femD_1 femD_2 GDPk_1 glmU_1 glmU_2 GLUCextI GLUextI gpdA_1 guaA_1 guaB_1 hasC_1 hisA_1 hisB_1 hisC_1 hisD_1 hisG_1 hisH_1 hisI_1 hisI_2 hisK_1 ilvB_2 ilvC_2 ilvD_2 leuA_1 leuB_1 leuC_1 leuD_1 LIPass lump_1 lump_2

lump_3 lump_4 lysA_1 METextI mraY_1 murB_1 murC_1 murD_1 murE_1 murF_1 murG_1 murI_1 pepV_1 pfk_1 pfl_1 pgk_1 pgsA_1 pgsA_2 pheA_1 pMET_1 proA_1 proB_1 proC_1 PROTass purH_1 purH_2 pycA_1 pyrH_1 racD_1 rmlA_1 rmlB_1 rmlC_1 RNAass rpiA_1 SLFextI TDPk_1 thyA_1 UDPk_1 unk_18 unk_31 unk_32 unk_42 unk_43 unk_44 unk_45a unk_45b unk_46 unk_47 unk_5 unk_61 unk_62 unk_63 unk_64 yafB_1 ychH_1 ywiC_1

MOMA: adhA_1 adhE_1 adk_1 ARGextI aroA_1 aroB_1 aroC_1 aroD_1 aroE_1 aroK_1 BIOMass bPOLYS c140s_1 c141s_1 c160s_1 c161s_1 c180s_1 c181s_1 cdsA_1 cdsA_2 chem_2 CO2extO cpsM_1 cysE_1 d190s_1 dal_1 dapA_1 dapB_1 ddl_1 dfrA_1 dgkA_1 DIPHOS DNAass ETOHextO fabD_1 fabH_1 fbaA_1 femD_1 femD_2 FORMextO GDPk_1 glmU_1 glmU_2 GLUCextI GLUextI gpdA_1 guaA_1 guaB_1 hasC_1 hisA_1 hisB_1 hisC_1 hisD_1 hisG_1 hisH_1 hisI_1 hisI_2 hisK_1 ilvB_2 ilvC_2 ilvD_2 leuA_1 leuB_1 leuC_1 leuD_1 LIPass lump_1 lump_2 lump_3 lump_4 lysA_1 METextI mraY_1 murB_1 murC_1 murD_1 murE_1 murF_1 murG_1 murI_1 pCO2_1 pepV_1 pETOH_1 pfk_1 pfl_1 pFORM_1 pgiA_1 pgk_1 pgsA_1 pgsA_2 pheA_1 pMET_1 proA_1 proB_1 proC_1 PROTass purH_1 purH_2 pycA_1 pyrH_1 racD_1 rmlA_1 rmlB_1 rmlC_1 RNAass rpiA_1 SLFextI TDPk_1 thyA_1 tpiA_1 UDPk_1 unk_1 unk_18 unk_31 unk_32 unk_42 unk_43 unk_44 unk_45a unk_45b unk_46 unk_47 unk_5 unk_61 unk_62 unk_63 unk_64 yafB_1 ychH_1 ywiC_1 zwf_1

Aerobic, Rich Media

FBA: adk_1 ALAextO ARGextI BIOMass bPOLYS c140s_1 c141s_1 c160s_1 c161s_1 c180s_1 c181s_1 cdsA_1 cdsA_2 cpsM_1 CYSextO d190s_1 dal_1 ddl_1 dfrA_1 dgkA_1 DIPHOS DNAass fabD_1 fabH_1 fbaA_1 femD_1 femD_2 glmU_1 glmU_2 glyA_1 gpdA_1 hasC_1 HISextO LIPass lump_1 lump_2 lump_3 lump_4 LYSextO METextI mraY_1 murB_1 murC_1 murD_1 murE_1 murF_1 murG_1 murI_1 pfk_1 pgk_1 pgsA_1 pgsA_2 pMET_1 PROextO PROTass pyrH_1 racD_1 rmlA_1 rmlB_1 rmlC_1 RNAass rpiA_1 TDPk_1 thyA_1 UDPk_1 unk_42 unk_43 unk_44 unk_45a unk_45b unk_46 unk_47

MOMA: adhA_1 adhE_1 adk_1 ALAextO ARGextI BIOMass bPOLYS c140s_1 c141s_1 c160s_1 c161s_1 c180s_1 c181s_1 cdsA_1 cdsA_2 CO2extO cpsM_1 CYSextO d190s_1 dal_1 ddl_1 dfrA_1 dgkA_1 DIPHOS DNAass ETOHextO fabD_1 fabH_1 fbaA_1 femD_1 femD_2 glmU_1 glmU_2 GLUCextI glyA_1 gpdA_1 hasC_1 HISextO LIPass lump_1 lump_2 lump_3 lump_4 LYSextO METextI mraY_1 murB_1 murC_1 murD_1 murE_1 murF_1 murG_1 murI_1 pCO2_1 pdhC_1 pdhD_1 pETOH_1 pfk_1 pgiA_1 pgk_1 pgsA_1 pgsA_2 pMET_1 PROextO PROTass pyk_1 pyrH_1 racD_1 rmlA_1 rmlB_1 rmlC_1 RNAass rpiA_1 TDPk_1 thyA_1 tpiA_1 UDPk_1 unk_42 unk_43 unk_44 unk_45a unk_45b unk_46 unk_47

Aerobic, Minimal Media

FBA: adk_1 ARGextI aroA_1 aroB_1 aroC_1 aroD_1 aroE_1 aroK_1 BIOMass bPOLYS c140s_1 c141s_1 c160s_1 c161s_1 c180s_1 c181s_1 cdsA_1 cdsA_2 chem_2 cpsM_1 cysE_1 d190s_1 dal_1 dapA_1 dapB_1 ddl_1 dfrA_1 dgkA_1 DIPHOS DNAass fabD_1 fabH_1 fbaA_1 femD_1 femD_2 folD_1 folD_2 GDPk_1 glmU_1 glmU_2 GLUCextI GLUextI glyA_1 gpdA_1 guaA_1 guaB_1 hasC_1 hisA_1 hisB_1 hisC_1 hisD_1 hisG_1 hisH_1 hisI_1 hisI_2 hisK_1 ilvB_2 ilvC_2 ilvD_2 leuA_1 leuB_1 leuC_1 leuD_1 LIPass lump_1 lump_2 lump_3 lump_4 lysA_1 METextI mraY_1 murB_1 murC_1 murD_1 murE_1 murF_1 murG_1 murI_1 O2extI pepV_1 pfk_1 pgk_1 pgsA_1 pgsA_2 pheA_1 pMET_1 pO2_1 proA_1 proB_1 proC_1 PROTass purH_1 purH_2 pycA_1 pyrH_1 racD_1 rmlA_1 rmlB_1 rmlC_1 RNAass rpiA_1 serA_1 serB_1 serC_1 SLFextI TDPk_1 thyA_1 tpiA_1 UDPk_1 unk_18 unk_31 unk_32 unk_42 unk_43 unk_44 unk_45a unk_45b unk_46 unk_47 unk_5 unk_61 unk_62 unk_63 unk_64 yafB_1 ychH_1 ywiC_1

MOMA: adhA_1 adhE_1 adk_1 ARGextI aroA_1 aroB_1 aroC_1 aroD_1 aroE_1 aroK_1 BIOMass bPOLYS c140s_1 c141s_1 c160s_1 c161s_1 c180s_1 c181s_1 cdsA_1 cdsA_2 chem_2 CO2extO cpsM_1 cysE_1 d190s_1 dal_1 dapA_1 dapB_1 ddl_1 dfrA_1 dgkA_1 DIPHOS DNAass ETOHextO fabD_1 fabH_1 fbaA_1 femD_1 femD_2 folD_1 folD_2 GDPk_1 glmU_1 glmU_2 GLUCextI GLUextI glyA_1 gpdA_1 guaA_1 guaB_1 hasC_1 hisA_1 hisB_1 hisC_1 hisD_1 hisG_1 hisH_1 hisI_1 hisI_2 hisK_1 ilvB_2 ilvC_2 ilvD_2 leuA_1 leuB_1 leuC_1 leuD_1 LIPass lump_1 lump_2 lump_3 lump_4 lysA_1 METextI mraY_1 murB_1 murC_1 murD_1 murE_1 murF_1 murG_1 murI_1 O2extI pCO2_1 pdhC_1 pdhD_1 pepV_1 pETOH_1 pfk_1 pgiA_1 pgk_1 pgsA_1 pgsA_2 pheA_1 pMET_1 pO2_1 proA_1 proB_1 proC_1 PROTass purH_1 purH_2 pycA_1 pyrH_1 racD_1 rmlA_1 rmlB_1 rmlC_1 RNAass rpiA_1 serA_1 serB_1 serC_1 SLFextI TDPk_1 thyA_1 tpiA_1 UDPk_1 unk_1 unk_18 unk_31 unk_32 unk_42 unk_43 unk_44 unk_45a unk_45b unk_46 unk_47 unk_5 unk_61 unk_62 unk_63 unk_64 yafB_1 ychH_1 ywiC_1 zwf_1
