# Supplementary material for: Modeling Lactococcus lactis using a genome-scale flux model
Source: BMC Microbiol. 2005 Jun 27;5:39. doi: 10.1186/1471-2180-5-39 (PMC1185544; doi:10.1186/1471-2180-5-39)
Supplement: Additional File 5 — Modeling the shift from homolactic ot heterolactic metabolism. These tables summarize the modeling procedure for inclusion of appropriate constrains when applying FBA to simulate anaerobic growth. [file 1471-2180-5-39-S5.pdf]

Table 5 - Model results for growth under anaerobic conditions, when maximizing for biomass formation. In grey: simulation results corresponding to the tuned model (simulations used in Figure 1). For these simulations, unique and multiple solutions were determined. Unique solution results are underlined, while fluxes corresponding to multiple solutions are indicated within a range.

	**NX1**[1]	**NX2**[2]	**NX3**[3]	**NX4**[4]	**NX5**[5]	**NX6**[6]	**NX7**[7]	**NX8**[8]	**NX9**[9]	**NX10**[10]
	(mmol gDW^{-1} h^{-1})									
**Glucose**	**13.6**	**24.6**	**24.6**	**18.0**	**13.6**	**24.6**	**18**	**13.6**	**10.0**	**7.0**
Lactate	0	0	0	0	0	33.3 - 44.2	23.0-23.3	0	0	0
Formate	24.4	41.3	18.1	21.4	24.4	0-2.1	9.7-9.8	24.4	18.1	12.9
Ethanol	12.7	1.2	22.4	16.5	12.3	0-3.2	4.9-5.0	12.3	8.9	6.1
Acetate	11.3	0	0	3.91	11.2	0–2.2	3.8-3.9	11.2	8.7	6.6
Acetaldehyde	0	39.1	0	0	0	0–3.2	0	0	0	0
Pyruvate	0	0	24.0	11.5	0	0	0	0	0	0
3MOB[11]	0.021	0	0.012	0	0.021	0-2.2	0-0.5	0.021	0.081	0.131
3MOP[12]	0.016	0.008	0.011	0.009	0.016	0.01-1.33	0.009-0.03	0.016	0.067	0.110
4MOP[13]	0.022	0.021	0.012	0.024	0.022	0.01-0.88	0.01-0.03	0.022	0.095	0.156
Phenyllactate	0.017	0.006	0.008	0.008	0.018	0.008-1.32	0.008	0.017	0.086	0.143
Methional	0.015	0.012	0.002	0.002	0.005	0.002	0.002	0.005	0.026	0.043
Glycerol-3-P	0	4.186	0	0	0	0-4.3	0	0	0	0
Glycerol	0	0.011	0	0	0	0	0	0	0	0
Indol-3-acetete	0	0	0	0	0	0	0	0	0	0
CO_2	1.03	3.29	2.38	1.08	1.01	1.13	1.05	1.01	0.69	0.43
	(h^{-1})									
Biomass	0.79	0.82	0.82	0.82	0.79	0.82	0.82	0.79	0.59	0.43
Carbon recovery (%)[14]	1.00	0.39	0.51	0.68	1.00	1.00	1.00	1.00	1.02	1.05

[1] Anaerobic conditions,TCA cycle functional. Glucose uptake rate set according to Novak, L. *et al.* (2000)

[2] Same as NX1, but different glucose uptake rate (as reported by Thomas, T.D., *et al.*, 1979)

[3] Same as NX2, but TCA cycle not functional.

[4,5] Same as NX3, but different glucose uptake rates.

[6,7] Same as NX3 and NX4, but flux through pyruvate-formate lyase constraint to 2.15 and 9.8, respectively, and *in silico* cell is not able to secrete pyruvate.

[8] Same as NX5, but *in silico* cell is not able to secrete pyruvate.

[9,10,] Same as NX8, but different growth rates.

[11] 3-methyl-2-oxobutanoate

[12] 3-methyl-2-oxopentanoate

[13] 4-methyl-2-oxopentanoate

[14] Carbon recovery on lactate, formate, ethanol, acetate, CO_2 and biomass from glucose. It was assumed that 43% of the carbon atoms found in the biomass stemmed from glucose [21]. Biomass yield on glucose was determined considering the calculated molecular weight for cell DW of 27.8 g / C-mol.

Table 6 - Model results for growth under anaerobic conditions, when minimizing for substrates. In grey: simulation results corresponding to the tuned model (simulations used in Figure 2). For these simulations, unique and multiple solutions were determined. Unique solution results are underlined, while fluxes corresponding to multiple solutions are indicated within a range.

	**NS1[1]**	**NS2[2]**	**NS3[3]**	**NS4[4]**	**NS5[5]**	**NS6[6]**	**NS7[7]**	**NS8[8]**	**NS9[9]**	**NS10[10]**	**NS11[11]**
**Biomass (h^{-1})**	**0.76**	**0.76**	**0.76**	**0.64**	**0.64**	**0.56**	**0.56**	**0.48**	**0.36**	**0.25**	**0.18**
						($mmol\ gDW^{-1}\ h^{-1}$)					
Glucose	12.7	12.7	18.1	10.8	15.4	9.5	13.6	9.4	5.9	4.1	3.3
Lactate	0	0	33.7	0	28.6	0	25.1	12.4	2.3	0.8	0
Formate	22.9	23.2	0	19.6	0	17.3	0	6.0	9.4	7.3	6.0
Ethanol	10.9	11.1	0	9.4	0	8.2	0	1.5	3.6	2.9	2.9
Acetate	10.4	10.6	0	8.9	0	7.9	0	3.0	4.7	3.7	2.8
CO_2	0.95	0.61	0.71	0.51	0.60	0.45	0.57	0.08	0.06	0.04	0.14
Alanine	0	0	0	0	0	0	0	0	0	0	0
Arginine	0.131	0.131	0.131	0.110	0.110	0.096	0.096	0.083	0.062	0.043	0.031
Aspartate	0	0	0	0	0	0	0	0	0	0	0
Asparagine	0.188	0.188	0.188	0.159	0.159	0.139	0.139	0.119	0.089	0.062	0.045
Cysteine	0.109	0.109	0.109	0.091	0.091	0.080	0.080	0.069	0.051	0.036	0.026
Glutamate	0	0	0-0.246	0	0-0.207	0	0-0.181	0-0.258	0-0.194	0-0.135	0
Glutamine	0.410	0.410	0.164-0.410	0.345	0.138–0.345	0.302	0.120-0.301	0-0.258	0-0.194	0-0.135	0.097
Glycine	0	0	0	0	0	0	0	0	0	0	0
Histidine	0.048	0.048	0.048	0.040	0.040	0.035	0.035	0.030	0.023	0.016	0.011
Isoleucine	0.195	0.195	0.195	0.164	0.164	0.144	0.144	0.123	0.092	0.064	0.046
Leucine	0.278	0.278	0.278	0.234	0.234	0.205	0.205	0.175	0.132	0.091	0.066
Lysine	0.320	0.320	0.320	0.270	0.270	0.236	0.236	0-0.202	0-0.151	0-0.105	0.076
Methionine	0.080	0.080	0.080	0.067	0.067	0.059	0.059	0.050	0.038	0.026	0.019
Phenylalanine	0	0	0	0	0	0	0	0	0	0	0
Proline	0.112	0.112	0.112	0.094	0.094	0.082	0.082	0.071	0.053	0.037	0.026
Serine	1.483	1.253	1.045	1.055	0.880	0.923	0.670	1.917-2.328	1.439-1.745	0.999-1.212	0.297
Threonine	0	0	1.132	0	0.953	0	0.734	0	0	0	0
Tryptophan	0	0.262	0 - 0.262	0.220	0 - 0.220	0.193	0-0.193	0-0.165	0-0.124	0 - 0.086	0 -0.062
Tyrosine	0.262	0	0 - 0.262	0	0 - 0.220	0.141	0-0.193	0-0.165	0-0.124	0 - 0.086	0-0.062
Valine	0	0.230	0.230	0.194	0.194	0.169	0.169	0.145	0.109	0.076	0.054
Carbon recovery (%)[12]	0.99	0.99	1.02	0.99	1.02	1.00	1.02	1.04	1.04	1.04	0.99

[1] Anaerobic conditions,TCA not cycle functional. Growth rate set according to Thomas, T.D., *et al.* (1979).

[2] Same condition as [1]. Valine, leucine and isoleucine cannot be synthesized, as most *L. lactis* strains are auxotrophic for those amino acids.
[3] Same as [2],but pfl_1 was constraint do zero to predict for the experimental yield of lactate in glucose (Y_{SL}).
[4,6] Same as [2], but different growth rates.
[5,7,8,9,10,11] Same as [2]. For different growth rates, pfl_1 was constraint to predict for Y_{SL}. *Growth rate (pfl_1 flux)*: 0.64 h^1 (0); 0.56 h^1 (0.1); 0.48 h^1 (5.5); 0.36 h^1 (9.0); 0.25 h^1 (7.0); 0.18 h^1 (5.899).
[12] Carbon recovery on lactate, formate, ethanol, acetate, CO_2 and biomass from glucose from glucose. It was assumed that 43% of the carbon atoms found in the biomass stemmed from glucose [21]. Biomass yield on glucose was determined considering the calculated molecular weight for cell DW of 27.8 g / C-mol
